# A Comprehensive Analysis of the Small GTPases Ypt7 Involved in the Regulation of Fungal Development and Secondary Metabolism in *Monascus ruber* M7

**DOI:** 10.3389/fmicb.2019.00452

**Published:** 2019-03-18

**Authors:** Jiao Liu, Ming Lei, Youxiang Zhou, Fusheng Chen

**Affiliations:** ^1^Institute of Quality Standard and Testing Technology for Agro-Products, Hubei Academy of Agricultural Sciences, Wuhan, China; ^2^Key Laboratory of Environment Correlative Dietology, College of Food Science and Technology, Huazhong Agricultural University, Wuhan, China

**Keywords:** Ypt7, *Monascus*, development, secondary metabolism, regulation

## Abstract

Ypts (*y*east *p*rotein *t*ransport*s*),also called as *r*as-*a*ssociated *b*inding GTPases (Rab), are the largest group of the small GTPases family, which have been extensively studied in model eukaryotic cells and play a pivotal role in membane trafficking, while this study showed potential regulation role of Ypts in fungi. One of Ypts, Ypt7 may be involved in fungal development and secondary metabolism, but the exact mechanism still exists a controversy. In current study, the functions of a *Monascus ypt7* homologous gene (*mrypt*7) from *Monascus ruber* M7 was investigated by combination of gene-deletion (Δ*mrypt*7), overexpression (M7::*PtrpC*-*mrypt*7) and transcriptome analysis. Results showed that the radial growth rate of Δ*mrypt*7 was significantly slower than *M. ruber* M7, little conidia and ascospores can be observed in Δ*mrypt*7, but the yield of intracellular secondary metabolites was dramatically increased. Simultaneously, the *mrypt*7 overexpression strain possessed similar capacity for sporulation and secondary metabolism observed in *M. ruber* M7. Transcriptome results further illustrated that *mrypt*7 could coordinate with numerous genes involved in the vegetative growth, conidiogenesis, secondary metabolism biosynthesis and transportation of *M. ruber* M7. Combined with the similar effect of Ypt7 homologs on other fungi, we propose that Ypt7 works more like a global regulatory factor in fungi. To our knowledge, it is the first time to investigate Ypt7 functions in *Monascus*. It could also improve the understanding of Ypt7 functions in fungi.

## Introduction

The largest subfamily of *ra*t *s*arcoma (Ras) superfamily, *r*as-*a*ssociated *b*inding GTPases (Rab), also called as Ypt (*y*east *p*rotein *t*ransport) and Sec (*sec*retion) (Gallwitz et al., 1983; Salminen and Novick, 1987), are involved in the membrane trafficking regulation in all eukaryotes (Maringer et al., 2016; Shinde and Maddika, 2016; Yun et al., 2016; Pfeffer, 2017). As key regulators of membrane transportation, Rab GTPases cycle between GTP-bound (active) and GDP-bound (inactive) conformations which stimulated by guanine nucleotide exchange factors (GEFs). Typical Rab GTPase possess several conserved functional regions, including phosphate/Mg2+ binding domain(PM), GTP/GDP binding domain (G), C-terminal isoprenylation region (C), and so on. For example, the G domain provides phosphate contacts and supplies a Ser/Thr site which is co-ordinated by the Mg^2+^ ion. The conserved molecular switch mechanism have detailed in some reviews (Lee et al., 2009; Pylypenko et al., 2018). The first Ypt was discovered in *Saccharomyces cerevisiae* (Schmitt et al., 1986; Pereira-Leal, 2008; Li and Marlin, 2015), and the succedent research results have showed that there are total 11 encoded Ypt proteins in *S. cerevisiae*, and each of which possesses distinctive function at a particular stage of the membrane transport pathway (Pereira-Leal, 2008; Li and Marlin, 2015). In animal, dozens of Rab/Ypt are proven to regulate vesicle trafficking among organelles (Ohbayashi and Fukuda, 2012; Li and Marlin, 2015; Mignogna and D'Adamo, 2017; Pfeffer, 2017). In plant, Ypts also are required for intracellular trafficking from the trans-Golgi-network to the plasma membrane and/or prevacuolar compartments (Yun et al., 2016; Tripathy et al., 2017). The more detailed Ypts roles for vesicle transports in animal and plant are summarized in the previous reviews (Stenmark, 2009; Ao et al., 2014).

In fungi, the number of Ypt family is stable from 7 to 12 Ypts, each of which may be responsible for a particular stage of the membrane transport pathway (Pereira-Leal, 2008; Li and Marlin, 2015). Among them, Ypt7 is proved as a key regulator of the material movement and transformation among cellular compartments through vacuolar biogenesis and fusion (Ohsumi et al., 2002; Kashiwazaki et al., 2009; Balderhaar et al., 2010; Wickner, 2010), and the Ypt7-mediated vacuolar fission and fusion are proved to be essential for maintaining stabilities of the cytosolic pH and osmolarity, and storing and transferring intermediary metabolites like mammalian lysosomes and plant vacuoles (Richards et al., 2010; de Marcos Lousa and Denecke, 2016; BasuRay et al., 2018), while some investigations have also showed that Ypt7 can influence fungal development and secondary metabolism. For example, the *ypt*7 gene deletion or overexpression can lead to the variances of conidiogenesis and metabolism in fungi (Chanda et al., 2009a; Xu et al., 2012; Li et al., 2015; Liu et al., 2015; Zheng et al., 2015). However, it is still unclear how Ypt7 regulates fungal development and secondary metabolism, and the relationship among Ypt7-mediated vacuolar changes and fungal development and secondary metabolism.

*Monascus* spp., as one of the important edible filamentous fungi, can produce many beneficial *s*econdary *m*etabolite*s* (SMs) including *Monascus p*igment*s* (Mps), *m*onacolin *K* (MK), γ-aminobutyric acid and so on (Patakova, 2013; Wu et al., 2013). As such, its fermented products, red yeast rice, also named as *Hongqu* in China have been used as food additives for more than 2,000 years (Chen et al., 2015). What's more, *Hongqu* has been permitted to use as a food supplement in USA from 1900s due to its cholesterol-lowering effects (Heber et al., 1999). The European Food Safety Authority (EFSA) also published a scientific opinion related to the daily dose of *Hongqu* containing MK (ESFA, 2011). Although *cit*rinin (CIT), a nephrotoxic toxin produced by some *Monascus* strains ever hampered *Hongqu* use, nowadays the control and elimination of CIT in *Hongqu* have successfully been solved by the strain screenings or molecular biological techniques (Shimizu et al., 2005; He and Cox, 2016).

There were 7 ypt homologous genes (*ypt*1*-ypt*7), which functions are predicted (Table S1), have been discovered in the genome of *Monascus ruber* M7. In current paper, the functions of *Monascus ypt7* (*mrypt*7) gene were investigated by combination of gene disruption, overexpression and transcriptome analysis. The results have revealed that besides the membrane trafficking regulation like other fungi, *mrypt*7 can also coordinate with numerous genes involved in the development and metabolism of *M. ruber* M7. Combined with Ypt7 functions in other fungi (Bouchez et al., 2015; Liu et al., 2015; Yang et al., 2018), we discuss and propose that Ypt7 works more like a global regulatory factor in fungi. To our knowledge, it is the first time to investigate Ypt7 functions in *Monascus* which could help us to improve the understanding of Ypt7 functions in fungi.

## Materials and methods

### Fungal Strains, Culture Media, and Growth Conditions

*M. ruber* M7 (CCAM 070120, Culture Collection of State Key Laboratory of Agricultural Microbiology, Wuhan, China), which can produce Mps and CIT, but no MK (Chen and Hu, 2005; Chen et al., 2017), was used to generate the *mrypt*7 deletion strain (Δ*mrypt*7) and overexpression strain (M7::*PtrpC*-*mrypt*7). The *p*otato *d*extrose *a*gar medium (PDA), *C*zapek *y*east extract *a*gar medium (CYA), *g*lycerol *n*itrate agar medium (*25*%) (G25N) and *m*alt extract *a*gar medium (MA) were utilized to observe the strains phenotypic characterization (He et al., 2013). Neomycin-resistant transformants were selected on PDA media containing 15 μg/mL G418 (Sigma-Aldrich, Shanghai, China). All strains were maintained on PDA slant at 28°C.

### Cloning and Sequence Analysis of *mrypt*7 in *M. ruber* M7

Ypt family genes in *M. ruber* M7 genome were blast from NCBI (http://blast.ncbi.nlm.nih.gov/Blast.cgi) (Table S1). Amino acid sequence encoded by *mrypt*7 was predicted using the SoftBerry's FGENESH program (http://linux1.softberry.com/). *mrypt*7 homology was compared with 283 fungi Ypt7 downloaded from NCBI to analyze their primary structural features (http://weblogo.threeplusone.com/create.cgi).

### Construction and Verification of *mrypt*7 Gene Deletion and Overexpression Strains

The construction and verification of *mrypt*7 gene deletion and overexpression strains were implemented according to the literature references (Shao et al., 2009; Liu et al., 2014). Briefly, the *mrypt*7 gene deletion cassette (5′UTR-*G418*-3′UTR) and *mrypt*7 gene overexpression cassette (5′UTR-*G418*-*PtrpC*-*mrypt*7-3′UTR) were constructed by double-joint PCR with the primers listed in Table S2 (Yu et al., 2004), and shown schematically in Figure S1. The *mrypt*7 gene deletion and overexpression vectors were formed, and transformed to *M. ruber* M7 using *Agrobacterium tumefaciens*-mediated transformation system to generate the *mrypt*7 gene deletion mutants (Δ*mrypt*7) and overexpression transformants (M7::*PtrpC*-*mrypt*7), respectively. PCR and southern blot were used to verify the *mrypt*7 gene deletion and overexpression strains.

### Phenotypic Characterization

*M. ruber* M7, Δ*mrypt*7 and M7::*PtrpC*-*mrypt*7 were cultivated on PDA, CYA, MA and G25N for 10 d at 28°C to observe phenotypic characterization. Besides, the three above-mentioned strains were incubated on PDA for 3 d at 28°C for vacuole morphological observation. For a better distinction, the normal vauoles were designated vacuoles (Va), while smaller vauoles were designated fragment vauoles (Fv) (Chanda et al., 2009b).

### Detection of Mps and CIT

One milliliter freshly harvested spores (10^5^ cfu/mL) of each strain were inoculated on PDA plate covered with cellophane membranes, and incubated at 28°C for 11 days, the samples were taken every 2 days from the 3rd day to the 11th day of culture to measure the Mps and CIT production. Freeze-dried mycelia and medium powder (0.1 g) was suspended in 1 mL 80 % (v/v) methanol solution, and subjected to 30 min ultrasonication treatment (KQ-250B, Kunshan, China).

The Mps and CIT were separated by an ACQUITY UPLC BEH C18 column (2.1 mm × 100 mm, 1.7 μm), and detected on Waters ACQUITY UPLC I-class system (Waters, Milford, MA, USA). A gradient elution was performed with the mobile phase including solvent A (0.1% formic acid in water) and solvent B (acetonitrile) with a flow rate of 0.3 mL/min and an injection volume of 2 μL. The gradient elution was performed as follows: 60% (v/v) solvent A with 40% (v/v) solvent B maintained for 0.5 min firstly, the content of solvent A was decreased from 60 to 20% for 21 min, and then from 20 to 60% for 0.5 min. Finally, the column was equilibrated with 60% solvent A for 3 min. The temperature of chromatographic column and samples were maintained at 40°C and 4°C, respectively.

### RNA Extraction, Library Preparation and Sequencing

Since *M. ruber* M7 and M7::*PtrpC*-*mrypt*7 shared similar phenotype and SMs yield (Figures 2, 3), the *mrypt*7 functions were further investigated only between *M. ruber* M7 and Δ*mrypt*7 by transcriptome analysis. Detailly, 1 mL freshly harvested spores (10^5^ cfu/mL) of *M. ruber* M7 and Δ*mrypt*7 were inoculated on PDA plate covered with cellophane membranes, and incubated at 28°C. Besides, based on our previous results, *Monascus ruber* M7 starts conidiation at 3rd day on PDA medium, and the secondary metabolited yield reached a relatively high level in 7th day, so the mycelium after cultured 3 days and 7 days were collected and used for the total RNA extraction by TRIzol Reagent (Invitrogen, Life Technologies, USA), two biological replicates were designed for each condition(Muraguchi et al., 2015; Srikumar et al., 2015; Heuston et al., 2018). The RNA purity and integrity were analyzed by Nanodrop NanoPhotometer spectrophotometer (NanoDrop products IMPLEN, CA, USA) and Agilent 2100 BioAnalyzer (Agilent, USA).

For each sample, the cDNA library was constructed using RNA Library Prep Kit for Illumina (NEB, USA). The obtained PCR products were purified by AMPure XP system and library quality was assessed on the Agilent Bioanalyzer 2100 system. The eight samples (M7-3d vs. M7-7d, Δ*mrypt*7-3d vs. Δ*mrypt*7-7d, M7-3d vs. Δ*mrypt*7-3d and M7-7d vs. Δ*mrypt*7-7d, with two repeats in each group) were sequenced using the BGIseq-500RS platform (BGI, Wuhan, China, http://www.mgitech.cn/product/detail/BGISEQ-500.html).

### Sequence Quality Evaluation and Validation

The obtained sequence raw reads of above-mentioned 8 samples were saved as FASTQ files, then the clean data were obtained after removing reads containing adapter, reads containing ploy-N and low quality reads from raw data. The expression levels of 10 randomly selected genes in *M. ruber* M7 were validated by qRT-PCR following the protocol of the RevertAid First Strand cDNA Synthesis Kit (Thermo Scientific, Japan) and the SYBR® Select Master Mix (ABI, USA).

### Functional Analysis of Transcriptome Data

The *M. ruber* M7 genome which contains 8,407 genes was used as a reference genome (Chen, 2015) to calculate the blast rate of genome and clean data by *H*ierarchical *I*ndexing for *S*pliced *A*lignment of *T*ranscripts (HISAT) and Bowtie2 (Langmead and Salzberg, 2012; Kim et al., 2015).

Gene expression levels were estimated by *R*NA-*S*eq by *E*xpectation-*M*aximization (RSEM), the normalized value of *f* ragments *p*er *k*ilobase of transcript per *m*illion mapped reads (FPKM) was used as a parameter to compare the expression levels between *M. ruber* M7 and Δ*mrypt*7(Li and Dewey, 2011; Van Verk et al., 2013). The orthologs with significantly different expression were identified by NOISeq method with an absolute value of log_2−_fold change >1 and probability >0.8 (Tarazona et al., 2012).

*G*ene *o*ntology (GO) (http://www.geneontology.org/) and KEGG pathway (http://www.kegg.jp/) function analysis were implemented to investigate the functions of the *d*ifferentially *e*xpressed *g*ene*s* (DEGs) between *M. ruber* M7 and Δ*mrypt*7. Moreover, the DEGs involved in fungal growth, sporation and secondary metabolism were further analyzed to illuminate the Mrypt7 role in fungal development and secondary metabolism.

## Results

### Sequence Analysis and Characterization of *mrypt*7 in *M. ruber* M7

The Ypt family genes in *M. ruber* M7 genome were blasted from NCBI, totally 7 Ypts showed highly homologous with other fungi (Table S1). Among them, Ypt7 homology was further analyzed in this study. Detailly, a 954 bp fragment containing the putative *mrypt*7 homolog was successfully amplified from the genomic DNA of *M. ruber* M7. A database searched with softberry (http://linux1.softberry.com/berry.phtml) has been showed that the CDS (*c*o*d*ing *s*equence) length of *mrypt*7 gene is 591 bp which encodes 196-amino acids and consists of 5 exons (Figure S2). The characteristic motifs or residues of Ypt7 from M7 and other 283 fungi downloaded from NCBI were investigated, and the results illustrated that *p*hosphate/*M*g^2+^ binding domain(PM), GTP/GDP binding domains (G) and C-terminal isoprenylation region (C) are highly conserved in all tested fungi (Figure S2). Besides, a database searched with NCBI-BLAST has been demonstrated that the deduced 196-amino acid sequences encoded by *mrypt*7 share 91% similarity with the GTP-binding protein Ypt7 of *Aspergillus fischeri* (Genbank: XP_001259484.1), *A. oryzae* (Genbank: XP_001824054.1), *A. niger* (Genban: XP_001398680.2), *P. oxalicum* (Genbank: EPS32522.1), and *P. zonata* (Genbank: XP_022585464.1) (Table S1).

### Verification of the *mrypt*7 Deletion and Overexpression Strains

**Figure 1:**
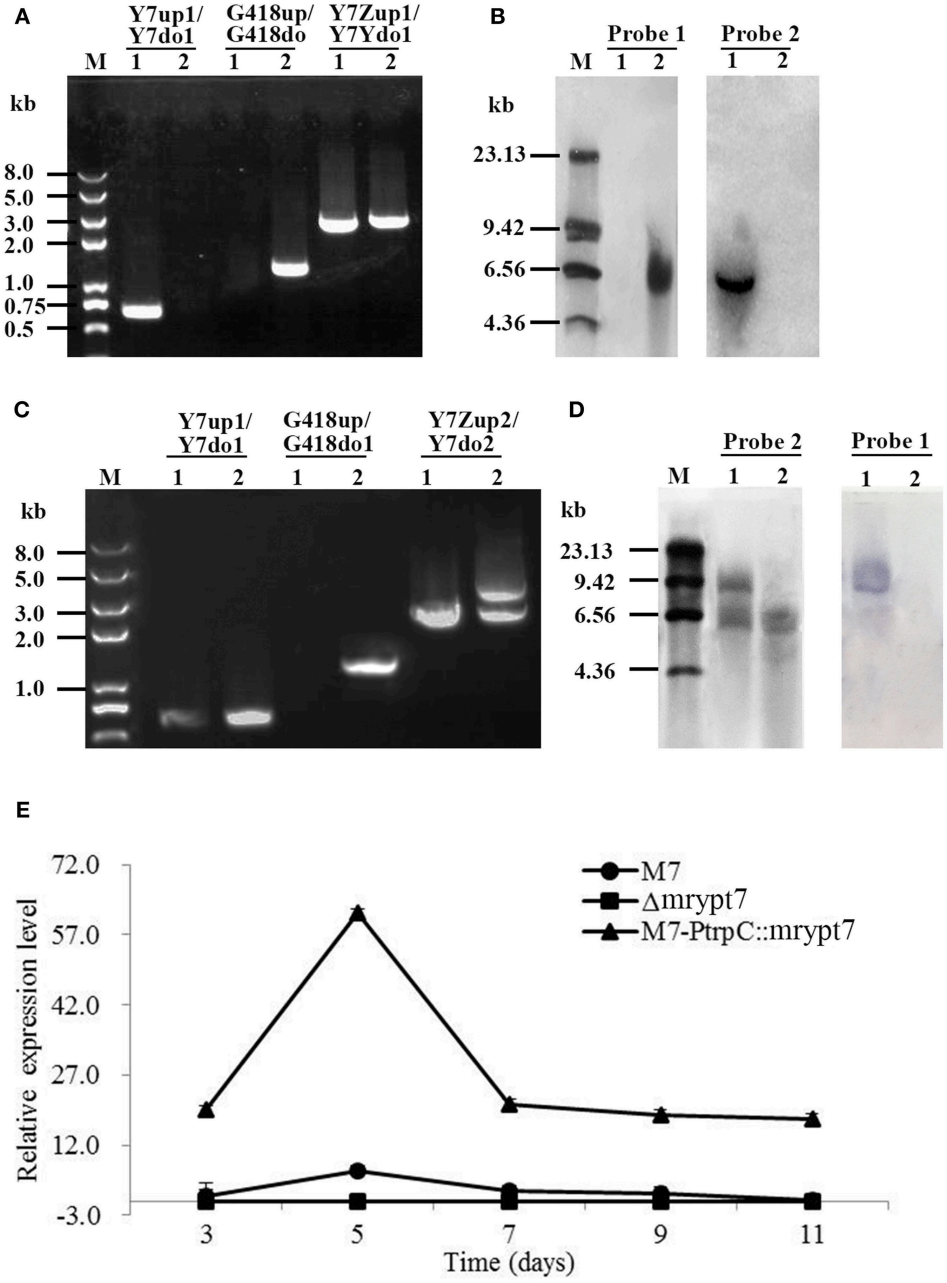
The verification of *mrypt*7 deletion and overexpression mutants. **(A)** PCR verification of the *mrypt*7 deletion mutant; **(B)** Southern blot verification of the *mrypt*7 deletion mutant; **(C)** PCR verification of the *mrypt*7 overexpression mutant; **(D)** Southern blot verification of the *mrypt*7 overexpression mutant; **(E)** qRT-PCR analysis of *mrypt*7 gene expression level.

Total 9 putative disruptants (Δ*mrypt*7) were obtained and analyzed, and one of them was displayed here. In PCR analysis as shown in Figure 1A, no DNA band was amplified when the genome of the putative Δ*mrypt*7 strain was used as template with the primer pair Y7-up1/Y7-do1 (Table S1), while a 0.7 kb product appeared using the genome of the wild-type strain *M. ruber* M7. A 1.2-kb fragment of *G418* gene could be amplified from Δ*mrypt*7 using primers G418up/G418do (Table S1), while nothing could be obtained from *M. ruber* M7. Meanwhile, amplicons of *M. ruber* M7 (2.3 kb) and Δ*mrypt*7 (2.4 kb) different in size were observed when primers Y7-Zup1/Y7-Ydo1 (Table S1) were used.

The putative Δ*mrypt*7 was further verified by Southern blot. As showed in Figure 1B, a probe corresponding to the *mrypt*7 coding region (probe 1,Table S2) yielded a single hybridizing band in a Southern blot of *Hin*dIII-digested genomic DNA of the wild-type strain, compared with no band in Δ*mrypt*7, which demonstrated that *M. ruber* M7 carried a single copy of *mrypt*7. Meanwhile, no band was detected in the wild-type strain, while a single band occurred in Δ*mrypt*7 using probe 2 (Table S2) which corresponds to the *G418* gene. These results proved that Δ*mrypt*7 carried a single integrated copy of the *mrypt*7 disruption cassette.

Total 16 putative M7::*PtrpC*-*mrypt*7 strains with G418 resistance were obtained and analyzed, and one of them was showed as follows. In PCR analysis as shown in Figure 1C, a 1.2-kb product appeared when the genome of the putative M7::*PtrpC*-*mrypt*7 strain was used as template with primers G418up/G418do (Table S2), while no DNA band was amplified using the genome of *M. ruber* M7. Amplicons of *M. ruber* M7 (3.0-kb) and M7::*PtrpC*-*mrypt*7 (4.5 kb and 3.0 kb) were totally different in size when primers Y7-up1/Y7-do1 (Table S2) was used, which proved that there were two copies of the *mrypt*7 overexpression cassette integrated in M7::*PtrpC*-*mrypt*7.

Southern blot analysis (Figure 1D) showed that probe 1 (Table S2) yielded two bands in M7::*PtrpC*-*mrypt*7 and a single band in *M. ruber* M7, while probe 2 (Table S2) generated a single band in M7:: *PtrpC*-*mrypt*7 and no band in *M. ruber* M7, which demonstrated that M7::*PtrpC*-*mrypt*7 carried two integrated copies of the *mrypt*7 and was a successful homologous recombination event.

qRT-PCR was implemented to analyze the transcription levels of the *mrypt*7 gene in *M. ruber* M7, Δ*mrypt*7 and M7::*PtrpC*-*mrypt*7. As shown in Figure 1E, Δ*mrypt*7 was deficient in the expression of the *mrypt*7 gene, the average level of *mrypt*7 expression in M7::*PtrpC*-*mrypt*7 was five times higher than that of *M. ruber* M7. These results further verified the success of gene knockout and overexpression in the putative Δ*mrypt*7 and M7::*PtrpC*-*mrypt*7 strains.

### Phenotypic Characterization of Δ*mrypt*7, M7::*PtrpC*-*mrypt*7 and *M. ruber* M7

**Figure 2:**
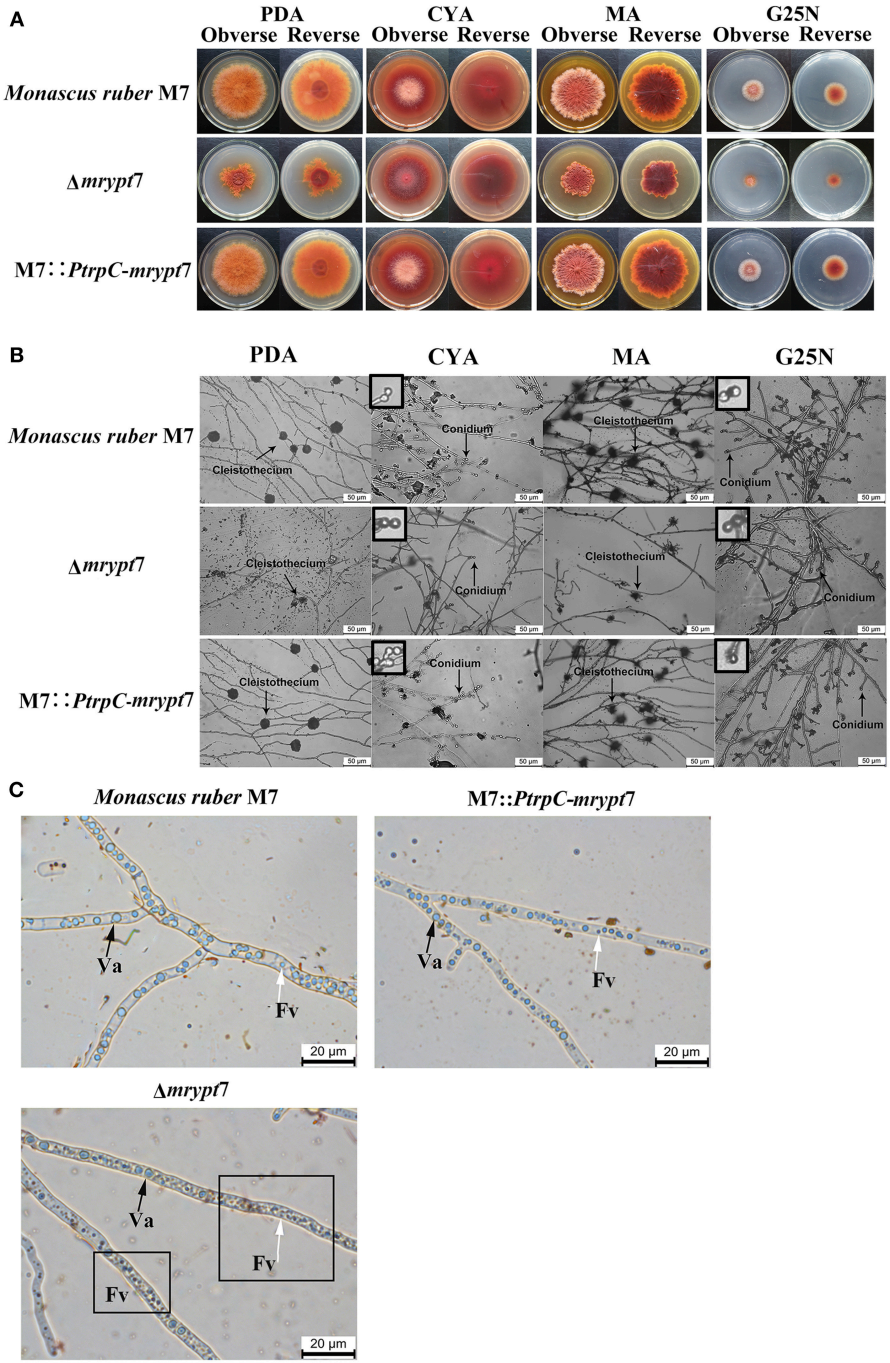
Phenotypic characterization of Δ*mrypt*7, M7::*PtrpC*-*mrypt*7 and *M. ruber* M7. **(A)** Colony morphologies on PDA, CYA, MA and G25N plates; **(B)** Microscopic structures on PDA, CYA, MA, and G25N plates, the enlarged area was indicated by arrow, size bar = 50 μm; **(C)** Vacuole and fragment vacuoles microscopic structures on PDA medium. Va, vacuole (black arrow); Fv, fragment vacuoles (white arrow).

Phenotypes of *Monacus ruber* were observed on the different media (PDA, CYA, MA, G25N) to investigate the influences of the *mrypt*7 deletion and overexpression on developmental processes. As showed in Figure 2A, the colony edge of Δ*mrypt*7 was irregular and the growth rates of Δ*mrypt*7 was slower than those of M7::*PtrpC*-*mrypt*7 and *M. ruber* M7. Besides, cleistothecia and conidia formation of Δ*mrypt*7 were obviously inhibited compared with M7::*PtrpC*-*mrypt*7 and *M. ruber* M7. While the colony phenotypes, growth rates and conidia formation of M7::*PtrpC*-*mrypt*7 had no significantly difference from those of *M. ruber* M7 (Figure 2B).

Vacuoles (Va) and fragment vacuoles (Fv) of *M. ruber* M7, Δ*mrypt*7 and M7::*PtrpC*-*mrypt*7 on PDA medium were also observed under microscope. Compared with M7::*PtrpC*-*mrypt*7 and *M. ruber* M7, the number of Fv in Δ*mrypt*7 increased more, while vacuoles reduced relatively and distributed nonuniformly in the mycelia (Figure 2C). The Fv and Va number and distribution between M7::*PtrpC*-*mrypt*7 and *M. ruber* M7 had no big difference, but the more uniform Fv and Va distribution of M7::*PtrpC*-*mrypt*7 was apparent (Figure 2C).

### Mps and CIT Production Analysis of Δ*mrypt*7, M7::*PtrpC*-*mrypt*7 and *M. ruber* M7

**Figure 3:**
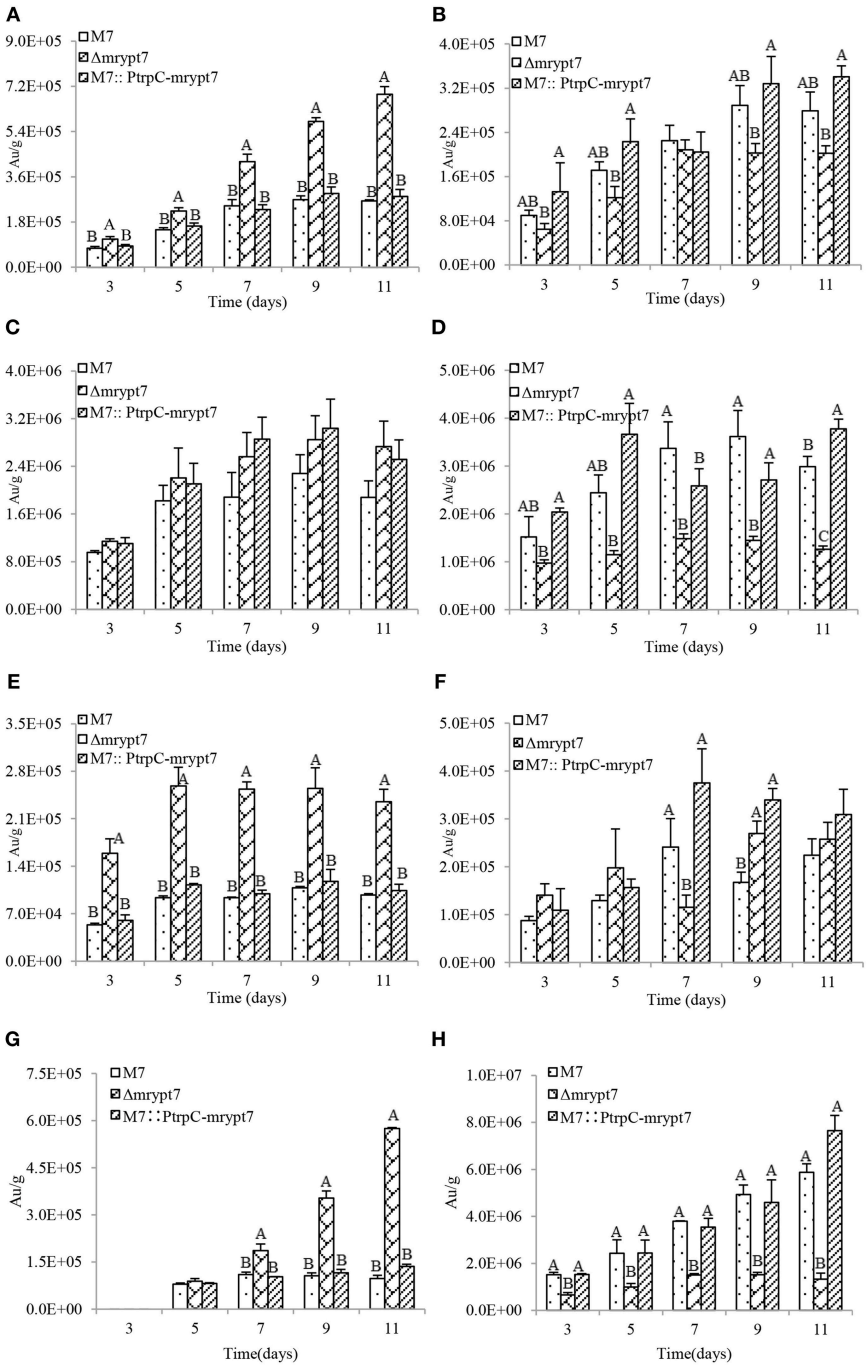
Mps and CIT yield analysis of *M.ruber* M7, Δ*mrypt*7 and M7::*PtrpC*-*mrypt*7. **(A)** The yield of intracellular Monasfloure A. **(B)** The yield of extracellular Monasfloure A. **(C)** The yield of intracellular Rubropunctatin. **(D)** The yield of extracellular Rubropunctatin. **(E)** The yield of intracellular Rubropunctamine. **(F)** The yield of extracellular Rubropunctamine. **(G)** The yield of intracellular CIT. **(H)** The yield of extracellular CIT. The error bar represents the standard deviation between the three repeats. Capitals signify *p*-value < 0.01.

Previous studies (Chen et al., 2017) have demonstrated that *M. ruber* M7 can produce Mps and CIT, but no MK, so the yields of the 8 main Mps (four yellow pigments, monasfloure A, monascine, monasflore B, ankaflavin; two orange pigments, rubropunctatin, monascuburin; two red pigments, rubropunctamine and monascuburamine) and CIT in *M. ruber* M7 and its mutants were analyzed in this study to uncover the effect of Mrypt7 on SMs. Generally, all the detected SMs were increased in the mycelia of Δ*mrypt*7 and M7::*PtrpC*-*mrypt*7, compared to *M. ruber* M7 (Figures S3, S4). Take monasfloure A, rubropunctatin, rubropunctamine and CIT production as examples for detail explanation, as showed in Figure 3, the concentration of intracellular yellow, orange and red pigments in Δ*mrypt*7 were 1.8 times, 1.3 times, and 2.8 times of those in *M. ruber* M7, respectively, while the production of extracellular yellow, orange and red pigments were 63, 45, and 83% of *M. ruber* M7. In contrast, both intracellular and extracellular Mps in M7::*PtrpC*-*mrypt*7 were increased at least 20% compared with *M. ruber* M7. The intracellular CIT concentration in Δ*mrypt*7 in 11th day was nearly 5 times more than those in M7::*PtrpC*-*mrypt*7 and *M. ruber* M7, while the extracellular CIT was only 20~40% of that in M7::*PtrpC*-*mrypt*7 and *M. ruber* M7. The intracellular and extracellular CIT in M7::*PtrpC*-*mrypt*7 and *M. ruber* M7 possessed the similar concentration.

### The Mrypt7 Function Elucidation on Development and Secondary Metabolite Production by Transcriptome Analysis

#### Differentially Expressed Genes Analysis, Annotation and Functional Classification

The transcriptome data obtained by RNA-seq were validated by qRT-PCR, β-actin serving as the reference gene. The expression data of 10 randomly selected genes (GME3693, GME5196, GME5065, GME2292, GME2157, GME5531, GME67, GME3412, GME6749, GME4561, GME2587) which are from the genome of *M. ruber* M7, fit with the sequencing profiles (Figure S5).

The *d*ifferentially *e*xpressed *g*ene*s* (DEGs) between M7-3d vs. M7-7d, Δ*mrypt*7-3d vs. Δ*mrypt*7-7d, M7-3d vs. Δ*mrypt*7-3d and M7-7d vs. Δ*mrypt*7-7d were analyzed. The DEGs' functions were analyzed through GO function classifications and KEGG pathway. According to GO categories, the DEGs function classifications of the four teams almost belong to biological process, cellular component and molecular function. KEGG pathway analysis manifested that the DEGs were mostly involved in cellular process, environmental information processing, genetic information processing, human diseases and metabolism. For example, the DEGs down-regulated in M7-3d vs. Δ*mrypt*7-3d included ankyrin repeat protein, G protein-coupled receptor and thiazole synthase, meanwhile, the DEGs up-regulated in M7-3d vs. Δ*mrypt*7-3d included syntaxin, Golgi SNAP receptor, Ras GTPase activating like protein and fungal type III polyketide synthase. The DEGs down-regulated in M7-7d vs. Δ*mrypt*7-7d included acyl-CoA synthetase, transposon, ubiquinone biosynthesis protein, exosome complex component and regulator of ribosome biosynthesis; while the DEGs up-regulated in M7-7d vs. Δ*mrypt*7-7d included golgi family apparatus membrane protein, mitochondrial fission protein, vesicular inhibitory amino acid molecule and gama tubulin complex.

#### The Fungus-Specific Regulators Coordinating Conidia Were Positively Regulated by Mrypt7

Fungal conidiation regulatory mechanism is very complex, and there are many regulators involved in fungal conidiation which can be divided into central regulators (brlA, abaA, and wetA), upstream activators (fluG, flbA, flbB, flbC, flbD, and flbE), negative regulators (CpcB, NsdC, NsdD, OsaA, SfgA, and VosA, etc.), velvet regulators (VeA, VelB, VelC, and VosA) and light responsive regulators (FphA, CryA, LreA, and LreB) (Park and Yu, 2012, 2016). The putative regulatory DEGs coordinating conidiation in M7 and Δ*mrypt*7 were analyzed in this part to illustrate the regulation of *mrypt*7 on *Monascus* conidia (Table 1).

**Table 1:** The putative regulatory DEGs involved in growth and conidiation in *M. ruber* M7.

**Regulators**		**Gene accession**	**Means**				Regulation^*^			
			**M7-3d**	**M7-7d**	**Δ*mrypt*7-3d**	**Δ*mrypt*7-7d**	**M7-3d vs. M7-7d**	**Δ*mrypt*7-3d vs. Δ*mrypt*7-7d**	**M7-3d vs. Δ*mrypt*7-3d**	**M7-7d vs. Δ*mrypt*7-7d**
Central regulators	brlA	GME2587	30.5	4.3	6.0	17.9	Down	Up	Down	Up
	abaA	**/**								
	wetA	GME2686	34.9	22.7	7.3	26.4	–	Up	Down	–
Upstream activators	flbA	GME7104	77.4	150.3	61.7	116.9	Up	–	–	–
	flbD	GME650	12.5	24.2	2.9	2.7	–	–	Down	Down
	fluG, flbB, flbC, flbE		No significant difference							
Negative activators	cpcB	GME2676	283.9	405.3	711.7	462.4	–	–	Up	–
	fadA	GME5261	110.0	188.7	84.2	166.6	–	Up	–	–
	nsdC	GME2944	96.8	184.4	96.9	191.2	Up	Up	–	–
	nsdD	GME7585	17.9	42.1	14.9	34.7	Up	Up	–	–
	sfaD	GME5747	69.8	151.8	77.8	114.7	Up	–	–	–
	ganB,gpgA,osaA,sfgD		No significant difference							
*velvet* regulators	veA	GME5196	78.3	52.2	83.8	162.2	–	Up	–	Up
	velB	GME7847	39.3	26.9	52.3	65.7	–	–	–	Up
	velC	/								
	vosA	GME6122	37.3	34.7	25.0	56.0	–	Up	–	–
Light responsive regulators	fphA	GME5823	0.2	0.1	0.3	0.1	–	–	–	–
	lreA	/								
	lreB	/								
	cryA	/								

* *Significantly different expression was identified by NOISeq method with an absolute value of log_2_-fold change >1 and Probability>0.8“Up” means the gene was up-regulated in the sample set*;

*“Down” means the gene was down-regulated in the sample set*;

*“-” means the gene possessed similar expression level in the sample set*;

*“/”means the gene had no homologs in M. ruber M7*.

The central regulatory pathway controls conidiation-specific gene expression and asexual developmental processes, very interesting, in *M. ruber* M7, the central regulatory pathway only includes *brlA* and *wetA* without *abaA* (Chen, 2015). Transcriptome results showed *brlA* and *wetA* were significantly down-regulated in Δ*mrypt*7-3d vs. M7-3d, even the *brlA* gene was up-regulated in Δ*mrypt*7-7d vs. M7-7d, the total expression levels of *brlA* in Δ*mrypt*7-3d and Δ*mrypt*7-7d were lower than those in *M. ruber* M7. Besides, the *flbD* gene belongs to one of the upstream developmental activators which is required for the initiation of conidiation and *brlA* activation (Kwon et al., 2010) was down-regulated in Δ*mrypt*7-7d. For balancing with upstream activators, on the contrast, the *cpcB* gene belongs to the negative regulator which inhibits precocious activation of *brlA* during proliferation (Park and Yu, 2012) was up-regulated in Δ*mrypt*7-3d. What's interesting is that the *velvet* regulators, *veA* and *velB* which suppresses conidiation and activation of sexual development (Bayram and Braus, 2012; Park and Yu, 2016) were up-regulated in Δ*mrypt*7-7d, but little cleistothecia could be found in Δ*mrypt*7 (Figure 2B), which was different from the results found in *Aspergillus nidulan* (Kim et al., 2002).

#### Effect of Mrypt7 on the Secondary Metabolites Biosynthesis Process

*Monascus* spp. can produce several secondary metabolites, like Mps, CIT, and so on (Liao et al., 2014; Feng et al., 2016). Previous studies have demonstrated that there are 9 predicted *pks* (*p*oly*k*etone *s*ynthase) genes in *M. ruber* M7 genome (Chen, 2015), and the different effects of Mrypt7 on these 9 *pks* genes were listed in Table 2. Among them, only the Mps *pks* was down-regulated in M7-3d vs. M7-7d, while all *pks* genes were up-regulated in Δ*mrypt*7-3d vs. Δ*mrypt*7-7d even only conidial yellow pigment *pks* and Mps *pks* reaching the significantly difference levels (log_2_-fold change>1 and probability>0.8). Besides, all *pks* genes down-regulated in M7-3d vs. Δ*mrypt*7-3d and only Mps *pks* gene and a putative lovastatin nonaketide *pks* gene reaching the significantly difference levels; while the putative lovastatin nonaketide synthase down-regulated and conidial yellow pigment, CIT and Mps *pks* up-regulated in M7-7d vs. Δ*mrypt*7-7d. Combination of these results has revealed that Mrypt7 can remarkably affect the expression of genes involved in SMs biosynthesis, but Mps and CIT *pks* genes may be more affected by Mrypt7.

**Table 2:** The putative 9 differential expression PKS genes in *M. ruber* M7.

**PKS ID**	**Homologs and related description**	**Evaluation index**	**M7-3d vs. M7-7d**	**Δ*mrypt*7-3d vs. Δ*mrypt*7-7d**	**M7-3d vs. Δ*mrypt*7-3d**	**M7-7d vs. Δ*mrypt*7-7d**
GME1661	Conidial yellow pigment biosynthesis polyketide synthase of *Byssochlamys spectabilis* No. 5	log_2_Ratio	0.91	6.48	−0.47	5.10
		Regulation	Up	Up^*^	Down	Up^*^
		Probability	0.45	0.99	0.23	0.98
GME2523	Similar to part of lovastatin diketide synthase from *Aspergillus terreus*	log_2_Ratio	1.61	1.16	−1.39	−1.84
		Regulation	Up^*^	Up	Down	Down
		Probability	0.81	0.25	0.28	0.58
GME2757	Similar to citrinin polyketide synthase of *Monascus purpureus*	log_2_Ratio	−0.87	2.31	−0.01	3.17
		Regulation	Down	Up^*^	Down	Up^*^
		Probability	0.51	0.94	0.02	0.93
GME4561	Similar to *Monascus* pigment biosynthesis polyketide synthase of *Monascus pilosus*	log_2_Ratio	−3.81	0.47	−1.32	2.96
		Regulation	Down^*^	Up	Down^*^	Up^*^
		Probability	0.98	0.87	0.96	0.98
GME6078	A putative polyketide synthase	log_2_Ratio	−0.46	0.78	−1.94	−0.71
		Regulation	Down	Up	Down	Down
		Probability	0.40	0.44	0.76	0.48
GME6749	Similar to putative lovastatin nonaketide synthase of *Glarea lozoyensis* 74030	log_2_Ratio	−0.18	0.04	−3.33	−3.11
		Regulation	Down	Up	Down^*^	Down^*^
		Probability	0.24	0.02	0.85	0.81
GME7032	Similar to lovastatin nonaketide synthase of *Fusarium oxysporum*	log_2_Ratio	−2.25	1.62	−2.36	1.51
		Regulation	Down	Up	Down	Up
		Probability	0.53	0.44	0.59	0.38
GME7327	A putative polyketide synthase	log_2_Ratio	−0.48	0.64	−0.18	0.94
		Regulation	Down	Up	Down	Up
		Probability	0.09	0.18	0.05	0.21
GME7426	A hybrid PKS-NRPS	log_2_Ratio	0.39	1.66	−0.61	0.66
		Regulation	Up	Up	Down	Up
		Probability	0.05	0.22	0.06	0.11

* *The DEGs reaching the significantly difference levels (log2-fold change >1 and probability>0.8)*.

Furture analysis of the expression level of Mps and CIT biosynthesis gene clusters showed that most of these genes down-regulated in M7-3d vs. Δ*mrypt*7-3d and up-regulated in M7-7d vs. Δ*mrypt*7-7d. Generally, for Mps biosynthesis gene cluster (Chen et al., 2017), the expressions of all genes (except *MpigH, MpigI*, and *MpigL*) were down-regulated in M7-3d vs. M7-7d, only *MpigH* and *MpigL* was up-regulated in Δ*mrypt*7-3d vs. Δ*mrypt*7-7d, the results suggested that the Mps biosynthesis gene cluster in Δ*mrypt*7 maintained a higher expression level compared with M7; nearly all genes down-regulated in Δ*mrypt*7-3d vs. M7-3d but only *MpigA, MpigC, MpigE, MpigF, MpigH, MpigL*, and *MpigN* reaching the significantly difference levels, on the contrary, the whole Mps gene cluster (except *MpigH* and *MpigI*) were up-regulated in Δ*mrypt*7-7d vs. M7-7d (Table 3).

**Table 3:** The differential expression of the Mps biosynthesis gene cluster genes in *M. ruber* M7.

**Gene ID**		**Function description**	**Up-down-regulation**			
			**M7-3d vs. M7-7d**	**Δ*mrypt*7-3d vs. Δ*mrypt*7-7d**	**M7-3d vs. Δ*mrypt*7-3d**	**M7-7d vs. Δ*mrypt*7-7d**
GME4561	MpigA	NR-PKS	Down^*^	Up	Down^*^	Up^*^
GME4562	MpigB	Transcription factor	Down^*^	Down	Down	Up^*^
GME4563	MpigC	C-11-Ketoreductase	Down^*^	Up	Down^*^	Up^*^
GME4564	MpigD	4-*O*-Acyltransferase	Down^*^	Down	Down	Up^*^
GME4565	MpigE	NAD(P)H-dependent oxidoreductase	Down^*^	Down	Down^*^	Up^*^
GME4566	MpigF	FAD-dependent oxidoreductase	Down^*^	Down	Down^*^	Up^*^
GME4567	MpigG	Serine hydrolase	Down^*^	Up	Down	Up^*^
GME4568	MpigH	Enoyl reductase	Up	Up^*^	Down^*^	Down
GME4569	MpigI	Transcription factor	Up	Up	Up	Up
GME4570	MpigJ	FAS subunit alpha	Down^*^	Up	Down	Up^*^
GME4571	MpigK	FAS subunit beta	Down^*^	Up	Down	Up^*^
GME4572	MpigL	Ankyrin repeat protein	Down	Up^*^	Down^*^	Up^*^
GME4573	MpigM	O-Acyltransferase	Down^*^	Up	Down	Up^*^
GME4574	MpigN	FAD-dependent monooxygenase	Down^*^	Up	Down^*^	Up^*^
GME4575	MpigO	Deacetylase	Down^*^	Down	Down	Up^*^
GME4576	MpigP	MFS multidrug transporter	Down^*^	Up	Down	Up^*^

* *The DEGs reaching the significantly difference levels (log2-fold change > 1 and probability > 0.8)*.

While for the CIT biosynthesis gene cluster (He and Cox, 2016), the expressions of *pksCT, MRL*7, *MRL*4, *MRL*2, *MRL*1, *MRR*2, and *MRR*2 down-regulated and *MRL*5, *MRR*4 up-regulated in M7-3d vs. M7-7d; *MRL*7, *MRL*6, *MRL*5, *MRL*4, *MRL*2, *MRL*1, *pksCT*, and *MRR*1 up-regulated and *MRR*2, *MRR*5 down-regulated in Δ*mrypt*7-3d vs. Δ*mrypt*7-7d; only *MRL*5 down-regulated and *MRR*3 up-regulated in Δ*mrypt*7-3d vs. M7-3d, but almost all genes (except *MRR*5) up-regulated in Δ*mrypt*7-7d vs. M7-7d (Table 4).

**Table 4:** The differential expression of the CIT biosynthesis gene cluster genes in *M. ruber* M7.

**Gene ID**		**Function description**	**Up-down-regulation**			
			**M7-3d vs. M7-7d**	**Δ*mrypt*7-3d vs. Δ*mrypt*7-7d**	**M7-3d vs. Δ*mrypt*7-3d**	**M7-7d vs. Δ*mrypt*7-7d**
GME2750	MRL7	Serine hydrolase	Down^*^	Up^*^	Up	Up^*^
GME2751	MRL6	Oxoglutarate/iron-dependent dioxygenase	Down	Up^*^	Down	Up^*^
GME2752	MRL5	Transcription factor	Up^*^	Up^*^	Down^*^	Up
GME2753	MRL4	Aldehyde dehydrogenase	Down^*^	Up^*^	Down	Up^*^
GME2754	MRL3	Aldoketomutase	Down	Up	Up	Up^*^
GME2755	MRL2	Dehydrogenase	Down^*^	Up^*^	Down	Up^*^
GME2756	MRL1	Glucose-methanol-choline oxidoreductase	Down^*^	Up^*^	Down	Up^*^
GME2757	pksCT	Citrinin PKS	Down	Up^*^	Down	Up^*^
GME2758	MRR1	MFS transporter	Up	Up^*^	Up	Up
GME2759	MRR2	Phosphoglycerate mutase	Down^*^	Down^*^	Up	Up
GME2760	MRR3	Hypothetical protein	Up	Up	Up^*^	Up
GME2761	MRR4	WD repeat protein	Up^*^	Up	Up	Up
GME2762	MRR5	Carbonic anhydrase	Down^*^	Down^*^	Down	Down^*^
GME2763	MRR6	Hypothetical protein	Down	Down	Up	Up
GME2764	MRR7	Enoyl reductase	Up	Up	Down	Down
GME2765	MRR8	Long-chain fatty acid transporter	Up	Up	Down	Up

* *The DEGs reaching the significantly difference levels (log2-fold change > 1 and probability > 0.8)*.

## Discussion

Ypt/Rab, a single-subunit small GTPase which is related in structure to the Gα subunit of heterotrimeric G proteins (large GTPases) (Santarpia et al., 2012), has been proved to be the key regulators of the membrane trafficking system, endocytosis and exocytosis in all eukaryotes, especially in animals and plants (Fu et al., 2017; Kim et al., 2017; Pfeffer, 2017; Srikanth et al., 2017). In fungi, the functions of Ypt/Rab, especially Ypt7, also only focus on its role of vesicle transport. It's an accepted fact that Ypt7 mainly controls vesicle–vacuolar fusion balance, the disruption and overexpression of Ypt7 caused various vacuole phenotypes (Xu et al., 2012; Li et al., 2015; Liu et al., 2015; Zheng et al., 2015). Besides, the mechanism of Ypt7 mediated fusion interacts with numerous tethering and SNARE (Soluble NSF attachment protein receptor) complexes had been proved (Balderhaar et al., 2010; Ng et al., 2012; Hyttinen et al., 2013). Moreover, conidiogenesis imperfection and SMs production variation can also be found in Ypt7 disruption or overexpression mutants (Chanda et al., 2009a; Li et al., 2015; Liu et al., 2015; Yang et al., 2018), but the mechanism of Ypt7 involved conidial biogenesis and SMs biosynthesis was unclear.

In current study, the functions of *mrypt*7 (*ypt*7 homologous) in *M. ruber* M7 were investigated, we have found that aside from the functions of vesicle fusion, Mrypt7 can synchronously regulate the vegetative growth, conidiogenesis and secondary metabolism in *M. ruber* M7. Transcriptome results illustrated that the fungus-specific conidiation regulators and SMs biosynthesis genes expression were significantly difference when *ypt*7 gene was deleted (Tables 1–4). So we propose that Ypt7 works more like a global regulatory factor in fungi, which is first put forward the novel function definition of Rab GTPases.

Fungal conidiation regulatory mechanism is very complex, and there are some differences for the regulatory gene distribution in different fungi. Compared to *A. nidulans*, the up-to-date regulatory genes were conserved in *M. ruber* M7, while no homolog hits of *abaA* (central regulators), *VelC* (velvet regulators), *CryA, LreA*, and *LreB* (light responsive regulators) were searched in *M. ruber* M7 (Table 1). It seems that a new regulatory network may be owned in *Monascus*. In current study, the *mrypt*7 disruption repressed asexual development, meanwhile, the regulators (*brlA, wetA* and *flbD*) related to conidia were down-regulated (Figure 2, Table 1), similar results were also found in other fungi like *Arthrobotrys oligospora* which was testified by qRT-PCR (Yang et al., 2018). These results suggest that Mrypt7 may be a positive regulator for *Monascus* asexual development and the relative regulation genes. While for *Monascus* sexual development process, the *mrypt*7-deletion promoted the expression level of *veA* and *velB*, but didn't activate sexual development as expected (Kim et al., 2002). The following tried to interpret a different sexual development regulation of *M. ruber* M7 may focus on the actual function of *veA* and *velB*, and try to find extra regulators coordinate to cleistothecia formation.

In this study, results indicated that the SMs biosynthesis was also regulated by Mrypt7. Transcriptome analysis showed that Mrypt7 had different impact on the expression of the 9 putative *pks* genes in *M. ruber* M7 Among them, Mps and CIT biosynthesis gene clusters and biosynthesis pathways have been delineated before (He and Cox, 2016; Chen et al., 2017), even both Mps and CIT in Δ*mrypt*7 were accumulated in the cell, the expression of Mps and CIT biosynthesis gene clusters showed variant expression level when the *mrypt*7 gene was deleted. Researches presented that vesicle localized enzyme were necessary for SMs biosynthesis until they were eventually turned over in vacuoles (Chanda et al., 2009b; Roze et al., 2011), based on the mycelial morphology and transcriptome results in this study, it's a reasonable statement that the level of the enzyme and the SMs production were affected by these relative genes expression level which regulated by Ypt7.

sBesides, it's proved that Rab/Ypt protein, SNARE, tethering factors and Sec1/Munc18-family protein worked together to mediate the intracellular destination of a transport vesicle (Baker et al., 2015; Milosevic and Sørensen, 2015; Baker and Hughson, 2016). In this study, four SNARE genes which were important for the transportation on Golgi and endosome (*bet1, bos1, stx16*, and *stx*7) and three tethering factors genes (golgins, vacuolar protein sorting 22 and transport protein particle complex 10) were differential expressed when *mrypt*7 gene was deleted (Table S3). Moreover, syntaxin 16 (Stx16), the important members of SNARE complex, which had been proved to mediate early/recycling endosome to trans-Golgi network and late endosome to trans-Golgi network traffic (Chen et al., 2010), was differential expressed when *mrypt*7 gene was deleted (Figure S6), these results suggest that Mrypt7 is functional in both in early and late endosomes. Mrypt7 and the above mentioned SNARE and tethering factor may work together to finish the fusion process and mediate SMs transportation. The further investigation of the interactions between these proteins could help to develop the detail model of Mrypt7 function in SMs transportation.

It's proved that Ypt family is stable from 7 to 12 Ypts in fungi, except Ypt7, others Ypts (Ypt2, Ypt5, Ypt6, etc.) also affect vegetative growth and conidiogenesis (Wakade et al., 2017; Yang et al., 2017), but had little influence on related genes (Yang et al., 2018). What's more, the Ypt7 disruption had no obvious effect on the expression of the rest of Ypts (Ypt1-Ypt6) in *M. ruber* M7, only Ypt3 was up-regulated in M7-7d vs. Δ*mrypt*7-7d (Table S3). The results further proved that Ypt7 worked more like a global regulator.

**Figure 4:**
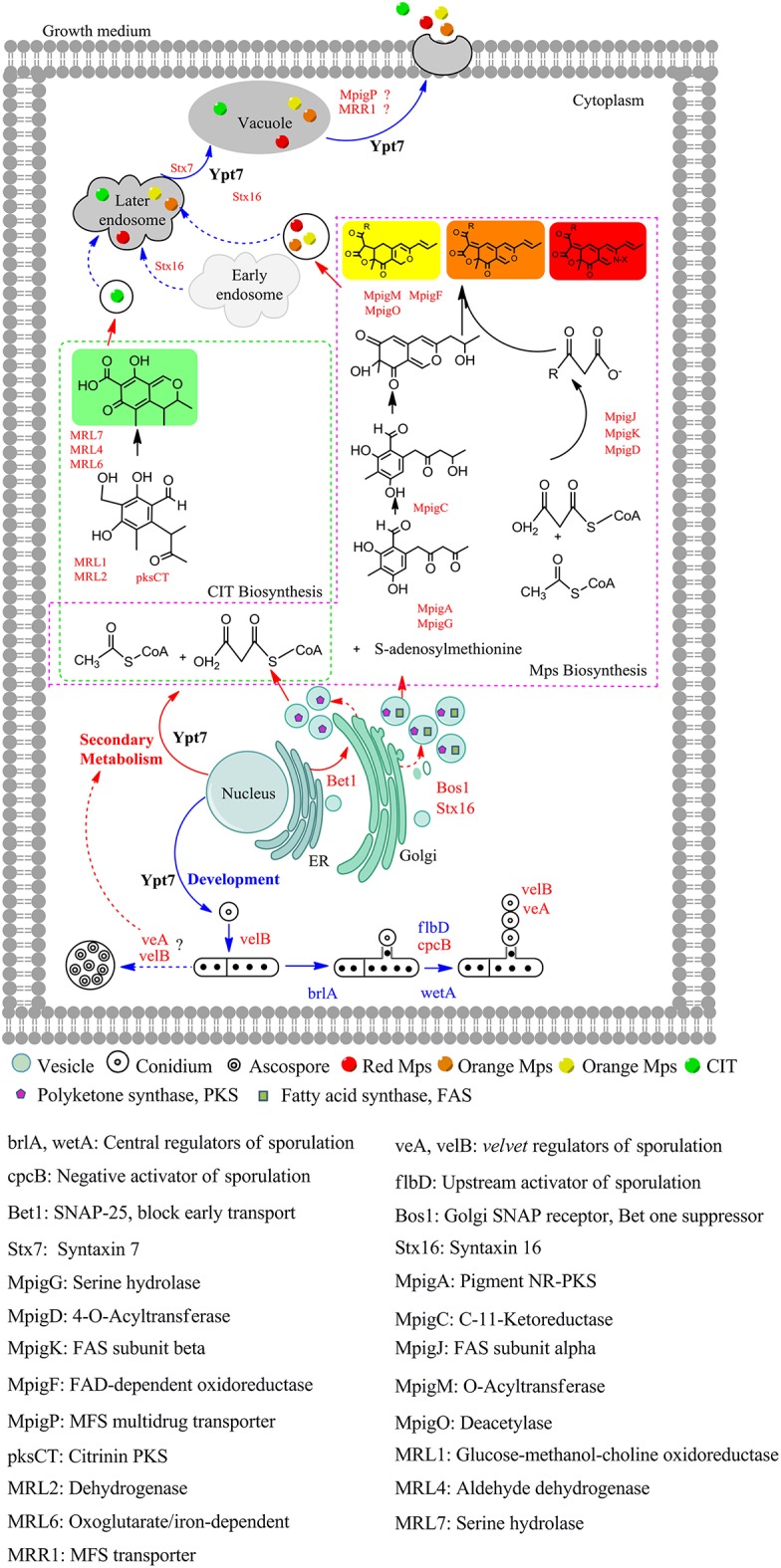
The proposed model of Ypt7 regulation physiological processes in fungi. The proteins and arrows marked in red indicating that they are up-regulated, the proteins and arrows marked in blue indicating that they are down-regulated, the proteins and arrows marked in black indicated the proved pathways. Dotted lines mean the supposed processes, solid lines mean the experimental processes in current study.

Based on the above results, a model of Ypt7 regulation physiological processes was proposed in this study (Figure 4). Briefly, Ypt7, a single-subunit small GTPase, worked as a global regulatory factor, is required for the development, secondary metabolism and vesicle fusion of *Monascus*. First, Ypt7 is a positive regulator for fungal development. The conidiogenesis is suppressed combined with the relative genes (*brlA, wetA, cpcB, flbD, veA*, and *velB*) differential expression when Ypt7 was deleted, more remarkable, the sexual development is still suppressed even the sexual active regulators (veA and velB) were up-regulated which suggested that the sexual development was more rely on the Ypt7 functional completeness (Yang et al., 2018). Besides, LaeA, the well-known global regulator, impacts asexual and sexual reproduction but has no noticeable effect on these genes (*brlA, wetA, cpcB, flbD, veA*, and *velB*) (Liu et al., 2016). Second, Ypt7 is a negative regulator for secondary metabolism, the SMs production remarkable rose when Ypt7 was deleted. Ypt7 disruption caused vesicles quantity significantly increased which may increase the vesicle localized SMs enzymes (Roze et al., 2011), and promoted the expression level of SMs biosynthesis gene (Yang et al., 2018). Third, Ypt7 regulates the early transport and later vesicle fusion simultaneously. the early transport between *e*ndoplasmic *r*eticulum (ER) and Golgi apparatus was effected by Ypt7 connecting with some SNARE genes, the up-regulate of Bet1 (*b*locked *e*arly *t*ransport) and Bos1 (*b*et one *s*uppressor) could help to alleviate the lethality associated with disruption of Ypt7 (Newman et al., 1990; Chung et al., 2018). The vesicle fusion and SMs secretion is hampered, but a small quantity of extracellular Mps and CIT can also be detected. Except the known role of Ypt7 in vesicle fusion, two up-regulated syntaxins (Stx7 and Stx16) (Chen et al., 2010) and up-regulated MpigP and MRR1 (multidrug transporters) were supposed to help Mps and CIT secretion.

In a conclusion, this study has indicated the effect and regulation model of *ypt*7 gene on vegetative growth, conidiogenesis, vesicle fusion and SMs biosynthesis and transportation in *Monascus*. This is the first comprehensive analysis of the Rab/Ypt family in *Monascus*, the results could enrich the understanding of the function of Rab/Ypt family and make some contribution to uncover the SMs biosynthesis and transportation process in filamentous fungi.

## Author Contributions

FC and YZ managed the project. JL and ML performed the transformants construction, secondary metabolites analysis and transcriptome results analysis in this work. JL performed the phenotypic characterization, interpreted the analysis results, and wrote the paper. All authors reviewed the manuscript.

### Conflict of Interest Statement

The authors declare that the research was conducted in the absence of any commercial or financial relationships that could be construed as a potential conflict of interest.

## Supplementary Material

Figure S1 The schematic diagram of the construction of *mrypt*7 deletion **(A)** and overexpression strains **(B)**.

Figure S2 Sequence analysis and characterization of *mrypt*7 in *M. ruber* M7. **(A)** Gene structure analysis by softbarry. CDSf, first (starting with start codon) coding segment; CDSi, internal (internal exon) coding segment; CDSl, last (ending with stop codon) coding segment; CDSo, gene contains the ONE coding exon only; PolA, terminal polyA signal; TSS, transcription start site. **(B)** Characteristic motifs or residues of Ypt7 in the choosed 285 fungi. phosphate/Mg^2+^ bingding domain(PM), GTP/GDP bingding domains **(G)** and C-terminal isoprenylation region **(C)**.

Figure S3 The mainly pigments of *M.ruber* M7 detected by UPLC. **(A)** The chromatogram of 4 main yellow pigments at 380 nm which are indicated by 1, 2, 3, and 4; **(B)** The chromatogram of 2 main orange pigments at 470 nm which are indicated by 5 and 6; **(C)** The chromatogram of the 2 main red pigments at 520 nm which are indicated by 7 and 8; **(D)** The chemical structure formula of the 8 pigments.

Figure S4 *Monascus* pigments yield analysis of *M.ruber* M7, Δ*mrypt*7 and M7::*PtrpC*-*mrypt*7. **(A)** The yield of intracellular Monasfloure A. **(B)** The yield of extracellular Monasfloure A. **(C)** The yield of intracellular Monascine. **(D)** The yield of extracellular Monascine. **(E)** The yield of intracellular Monasfluore B. **(F)** The yield of extracellular Monasfluore B. **(G)** The yield of intracellular Ankaflavin. **(H)** The yield of extracellular Ankaflavin. **(I)** The yield of intracellular Rubropunctatin. **(J)** The yield of extracellular Rubropunctatin. **(K)** The yield of intracellular Monascuburin. **(L)** The yield of extracellular Monascuburin. **(M)** The yield of intracellular Rubropunctamine. **(N)** The yield of extracellular Rubropunctamine. **(O)** The yield of intracellular Monascuburamine. **(P)** The yield of extracellular Monascuburamine. The error bar represents the standard deviation between the three repeats. Capitals signify *p*-value < 0.01.

Figure S5 The validation of transcriptome data by qRT-PCR. **(A)** The expression level of the ten selectived genes in M7-3d VS Δ*mrypt*7-3d. **(B)** The expression level of the ten selectived genes in M7-7d VS Δ*mrypt*7-7d. The expression level of the genes in M7 was set as 1.

Figure S6 The SNARE interactions in vesicular transport.

Table S1 Ypt homologous genes in the *M. ruber* M7 genome.

Table S2 Primers used for the deletion and overexpression of mrypt7 gene.

Table S3 The proposed genes involved in vesicle transport.
