# Supplementary figures and images for: A Comprehensive Analysis of the Small GTPases Ypt7 Involved in the Regulation of Fungal Development and Secondary Metabolism in Monascus ruber M7
Source: Front Microbiol. 2019 Mar 18;10:452. doi: 10.3389/fmicb.2019.00452 (PMC6431638; doi:10.3389/fmicb.2019.00452)

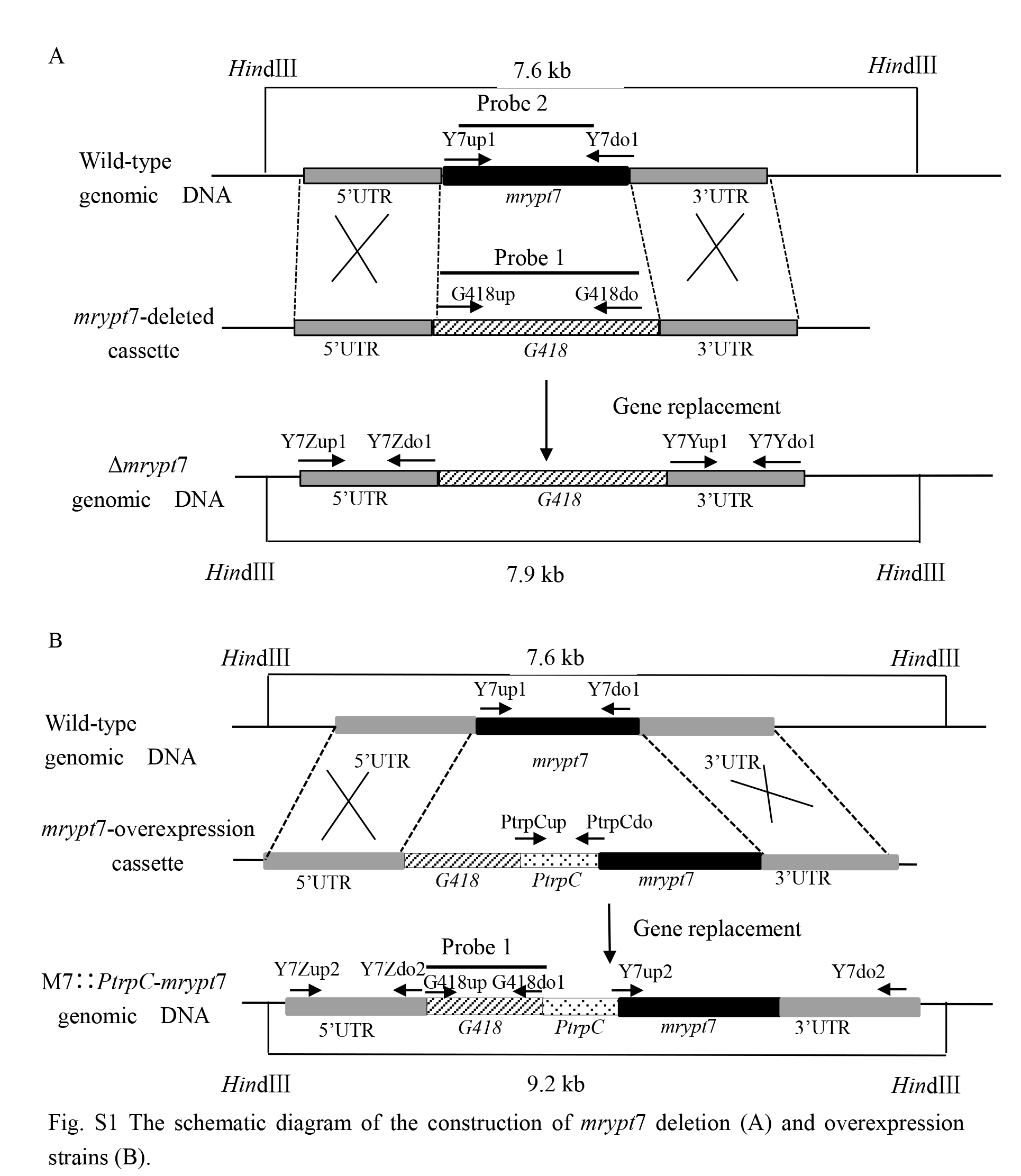

Supplement: Figure S1 — The schematic diagram of the construction of mrypt7 deletion (A) and overexpression strains (B). [file Image_1.TIF]

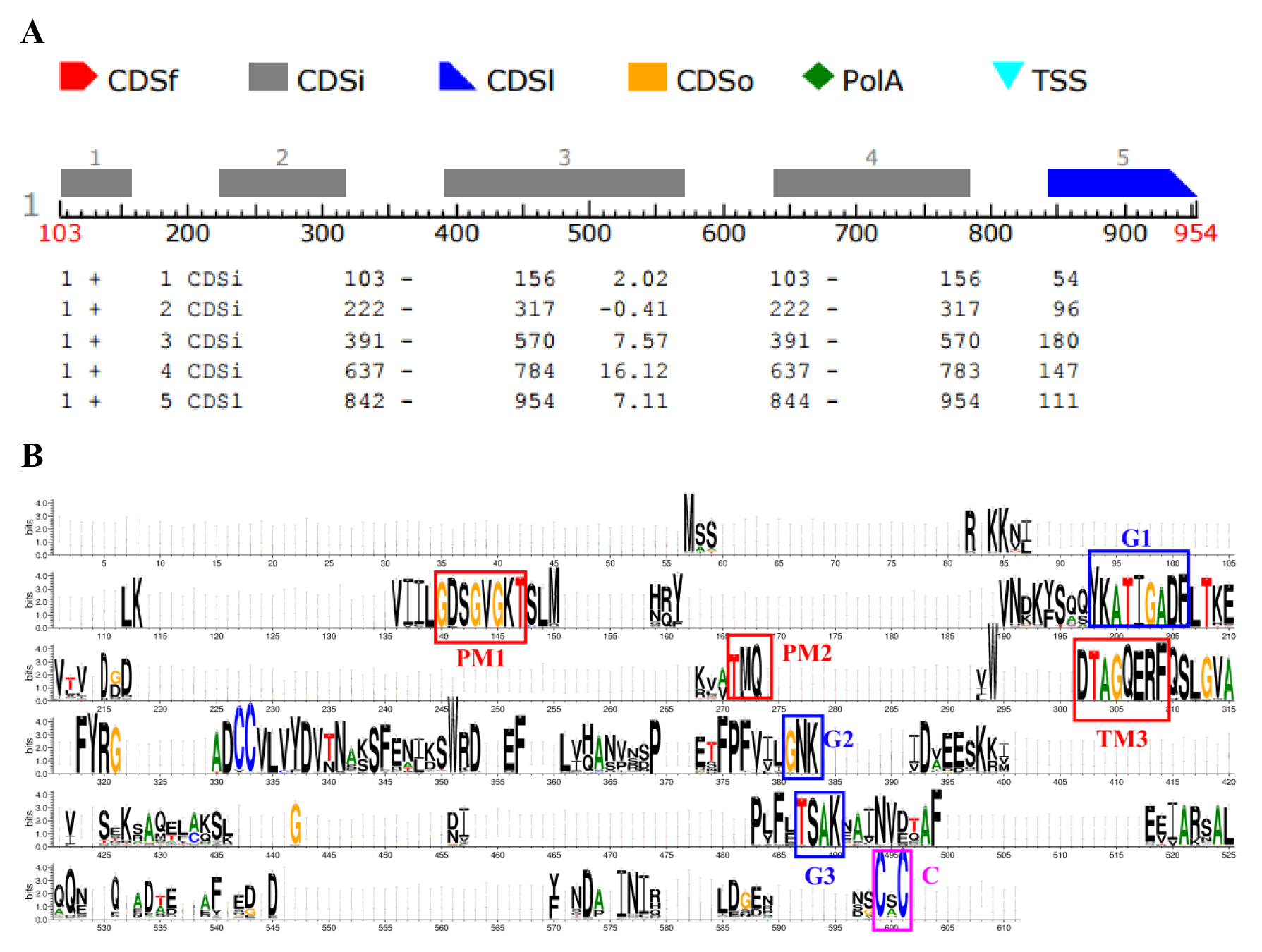

Supplement: Figure S2 — Sequence analysis and characterization of mrypt7 in M. ruber M7. (A) Gene structure analysis by softbarry. CDSf, first (starting with start codon) coding segment; CDSi, internal (internal exon) coding segment; CDSl, last (ending with stop codon) coding segment; CDSo, gene contains the ONE coding exon only; PolA, terminal polyA signal; TSS, transcription start site. (B) Characteristic motifs or residues of Ypt7 in the choosed 285 fungi. phosphate/Mg2+ bingding domain(PM), GTP/GDP bingding domains (G) and C-terminal isoprenylation region (C). [file Image_2.TIF]

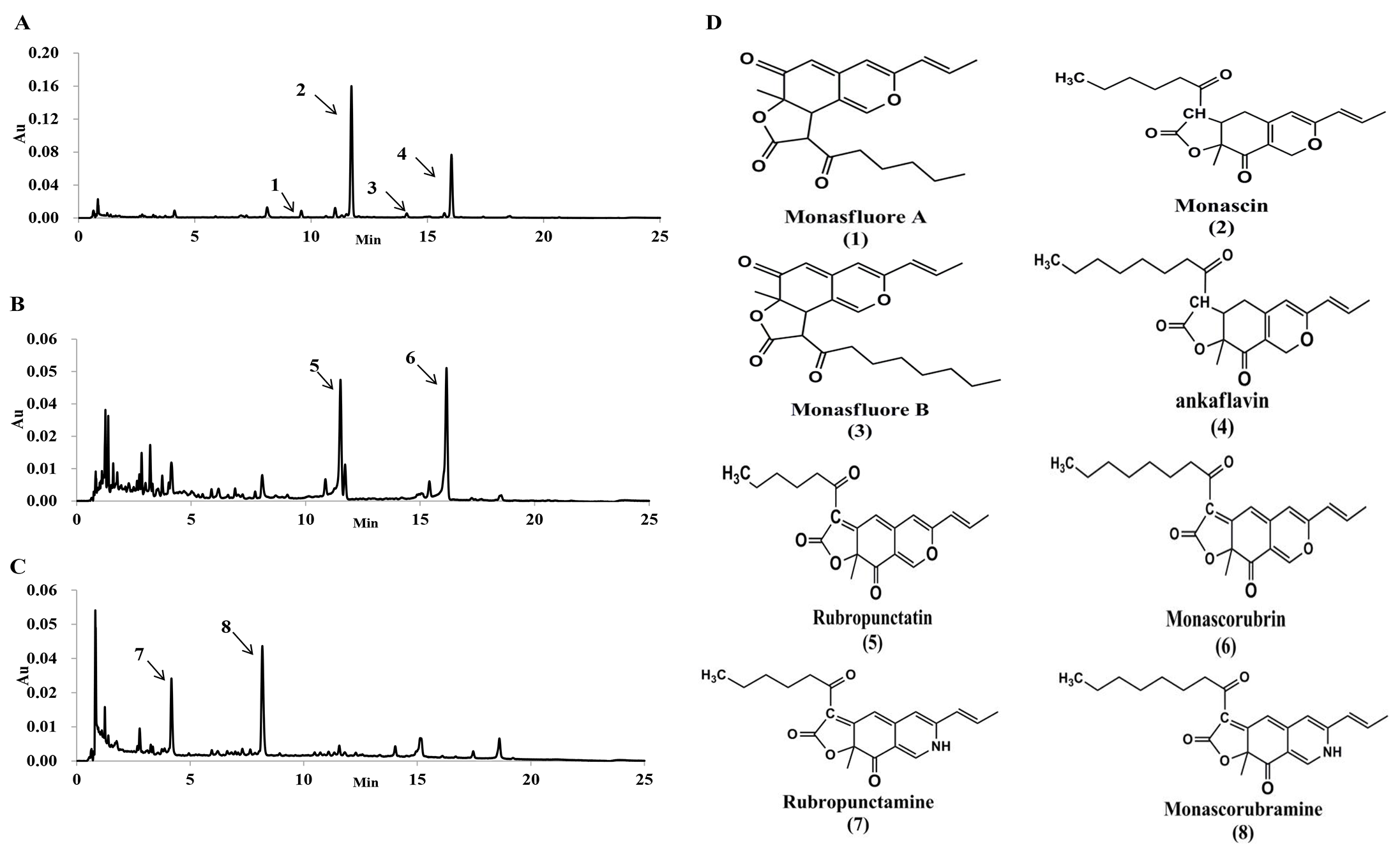

Supplement: Figure S3 — The mainly pigments of M.ruber M7 detected by UPLC. (A) The chromatogram of 4 main yellow pigments at 380 nm which are indicated by 1, 2, 3, and 4; (B) The chromatogram of 2 main orange pigments at 470 nm which are indicated by 5 and 6; (C) The chromatogram of the 2 main red pigments at 520 nm which are indicated by 7 and 8; (D) The chemical structure formula of the 8 pigments. [file Image_3.TIF]

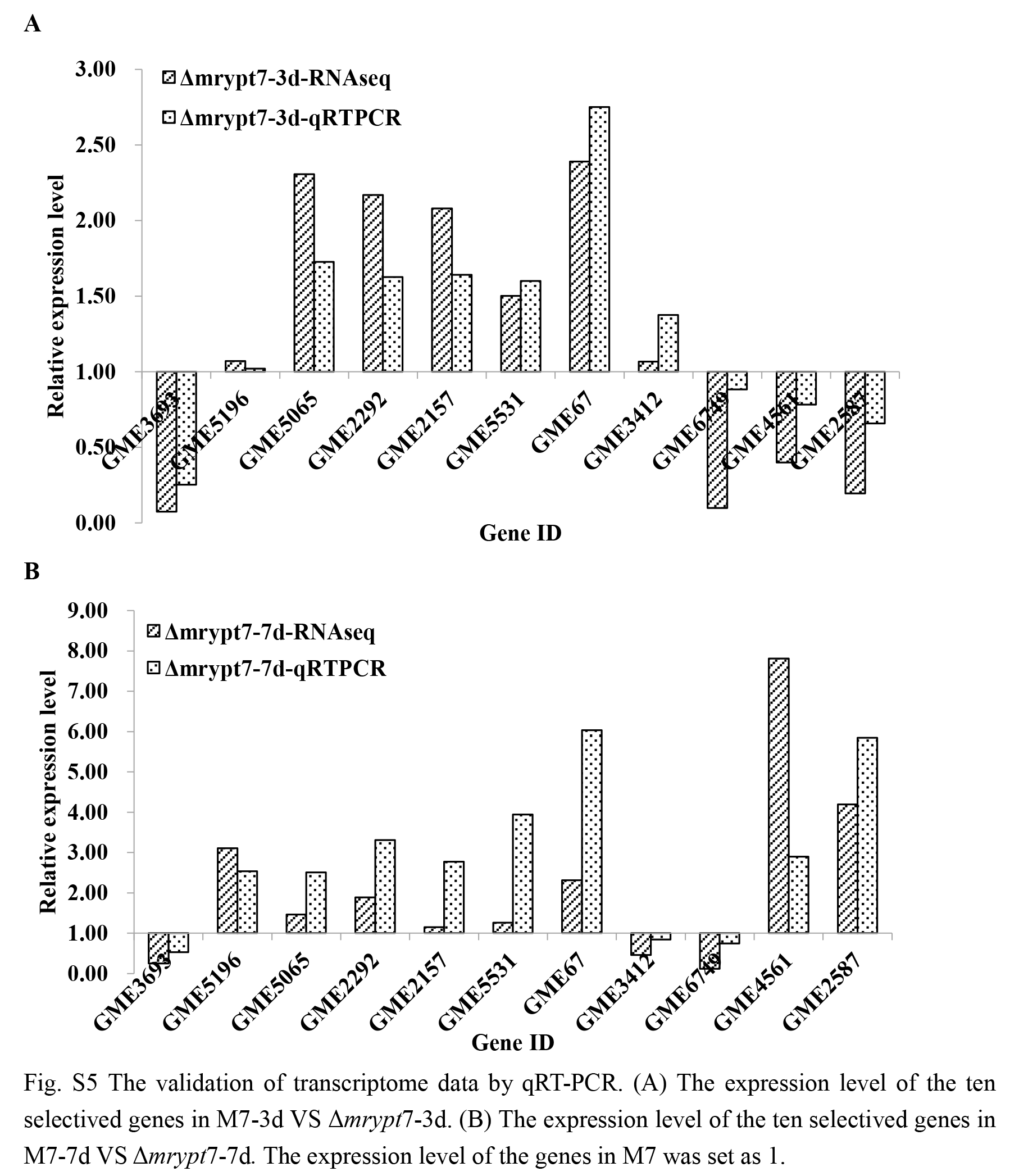

Supplement: Figure S5 — The validation of transcriptome data by qRT-PCR. (A) The expression level of the ten selectived genes in M7-3d VS Δmrypt7-3d. (B) The expression level of the ten selectived genes in M7-7d VS Δmrypt7-7d. The expression level of the genes in M7 was set as 1. [file Image_5.TIF]

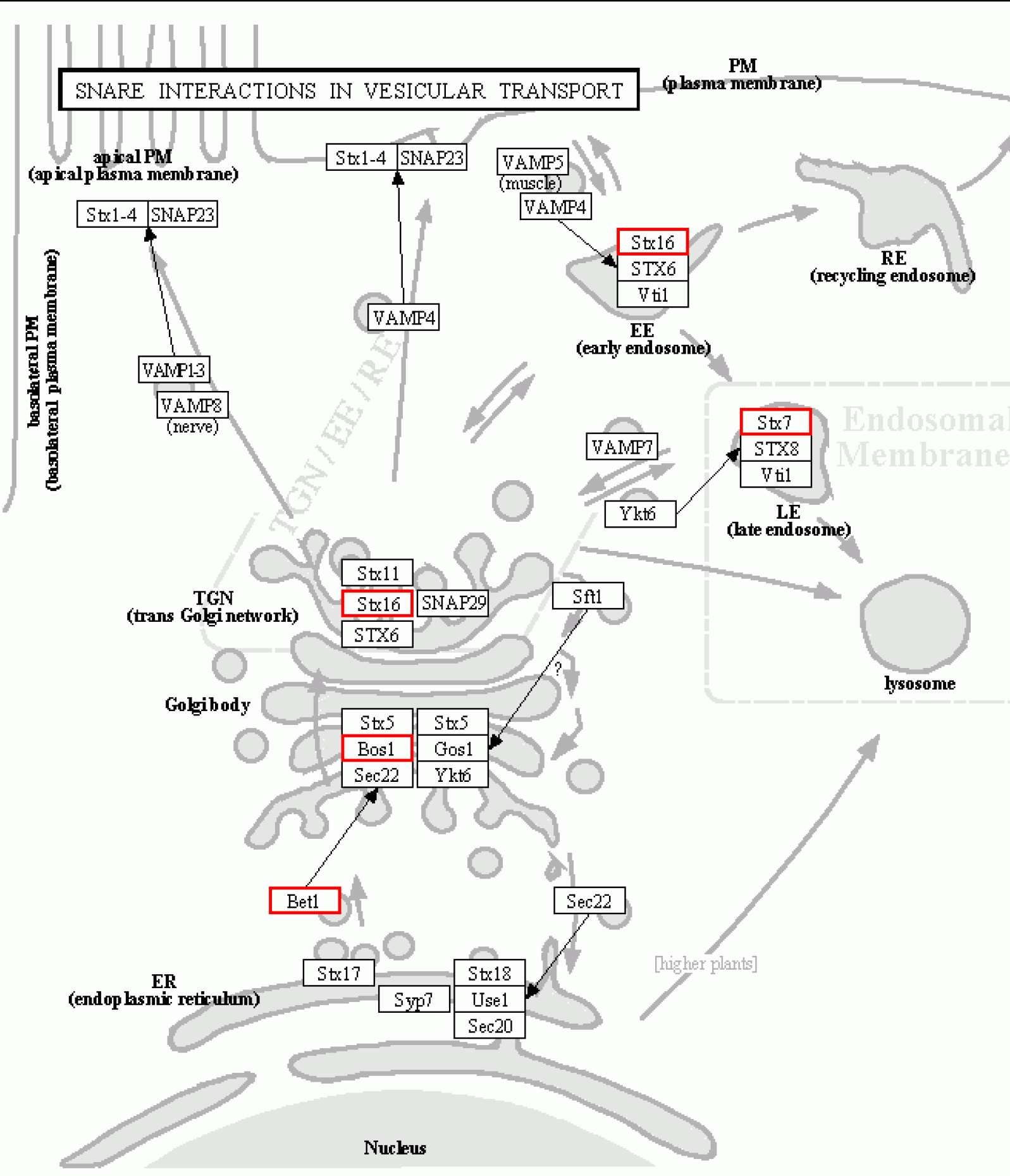

Supplement: Figure S6 — The SNARE interactions in vesicular transport. [file Image_6.TIF]
