# Supplementary material for: A Comprehensive Analysis of the Small GTPases Ypt7 Involved in the Regulation of Fungal Development and Secondary Metabolism in Monascus ruber M7
Source: Front Microbiol. 2019 Mar 18;10:452. doi: 10.3389/fmicb.2019.00452 (PMC6431638; doi:10.3389/fmicb.2019.00452)
Supplement: Figure S4 — Monascus pigments yield analysis of M.ruber M7, Δmrypt7 and M7::PtrpC-mrypt7. (A) The yield of intracellular Monasfloure A. (B) The yield of extracellular Monasfloure A. (C) The yield of intracellular Monascine. (D) The yield of extracellular Monascine. (E) The yield of intracellular Monasfluore B. (F) The yield of extracellular Monasfluore B. (G) The yield of intracellular Ankaflavin. (H) The yield of extracellular Ankaflavin. (I) The yield of intracellular Rubropunctatin. (J) The yield of extracellular Rubropunctatin. (K) The yield of intracellular Monascuburin. (L) The yield of extracellular Monascuburin. (M) The yield of intracellular Rubropunctamine. (N) The yield of extracellular Rubropunctamine. (O) The yield of intracellular Monascuburamine. (P) The yield of extracellular Monascuburamine. The error bar represents the standard deviation between the three repeats. Capitals signify p-value < 0.01. [file Image_4.pdf]

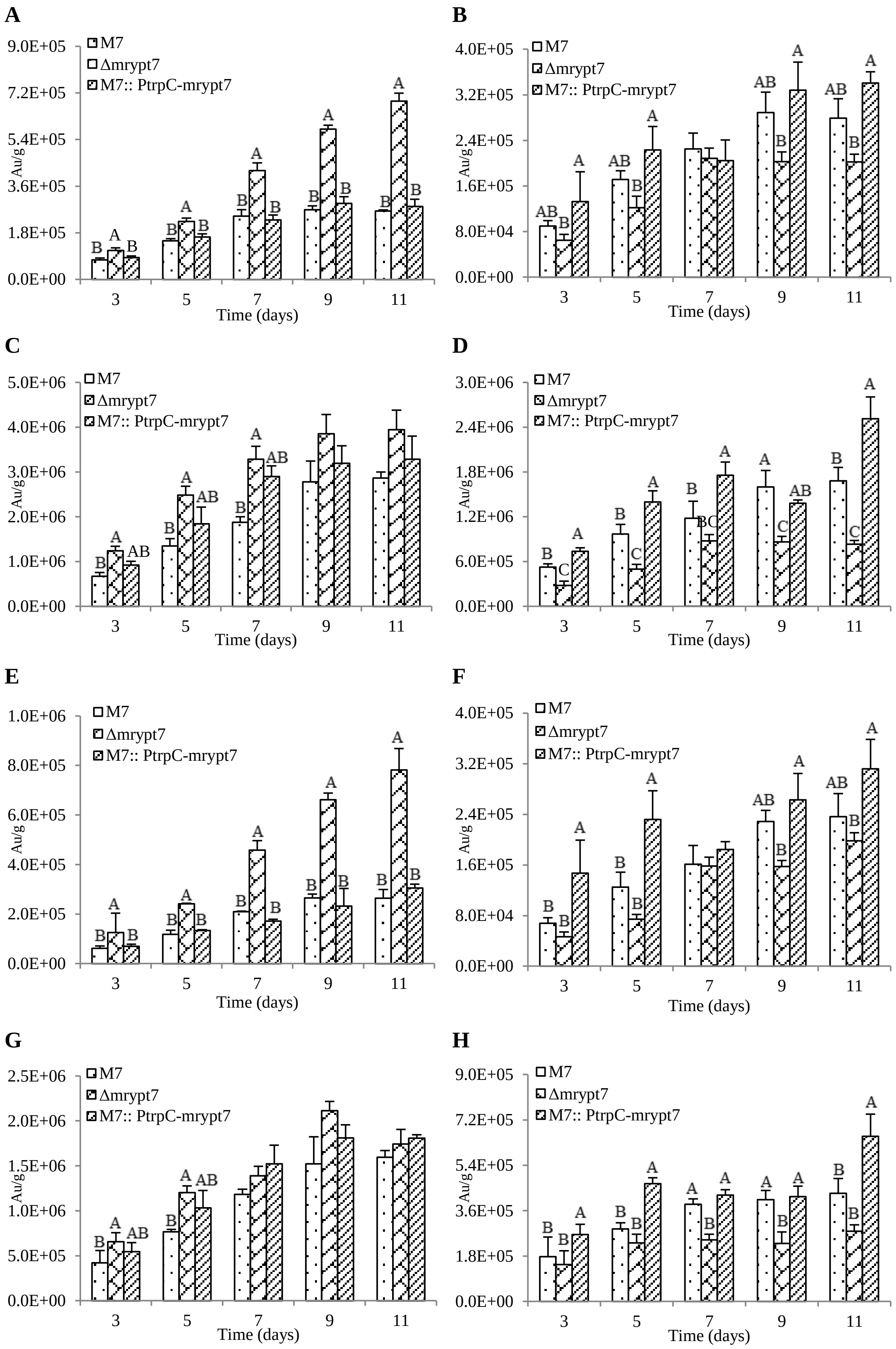
A
9.0E+05
7.2E+05
5.4E+05
3.6E+05
1.8E+05
0.0E+00
Au/g
M7
Δmrypt7
M7:: PtrpC-mrypt7
3
5
7
9
11
Time (days)
B
4.0E+05
3.2E+05
2.4E+05
1.6E+05
8.0E+04
0.0E+00
Au/g
M7
Δmrypt7
M7:: PtrpC-mrypt7
3
5
7
9
11
Time (days)
C
5.0E+06
4.0E+06
3.0E+06
2.0E+06
1.0E+06
0.0E+00
Au/g
M7
Δmrypt7
M7:: PtrpC-mrypt7
3
5
7
9
11
Time (days)
D
3.0E+06
2.4E+06
1.8E+06
1.2E+06
6.0E+05
0.0E+00
Au/g
M7
Δmrypt7
M7:: PtrpC-mrypt7
3
5
7
9
11
Time (days)
E
1.0E+06
8.0E+05
6.0E+05
4.0E+05
2.0E+05
0.0E+00
Au/g
M7
Δmrypt7
M7:: PtrpC-mrypt7
3
5
7
9
11
Time (days)
F
4.0E+05
3.2E+05
2.4E+05
1.6E+05
8.0E+04
0.0E+00
Au/g
M7
Δmrypt7
M7:: PtrpC-mrypt7
3
5
7
9
11
Time (days)
G
2.5E+06
2.0E+06
1.5E+06
1.0E+06
5.0E+05
0.0E+00
Au/g
M7
Δmrypt7
M7:: PtrpC-mrypt7
3
5
7
9
11
Time (days)
H
9.0E+05
7.2E+05
5.4E+05
3.6E+05
1.8E+05
0.0E+00
Au/g
M7
Δmrypt7
M7:: PtrpC-mrypt7
3
5
7
9
11
Time (days)

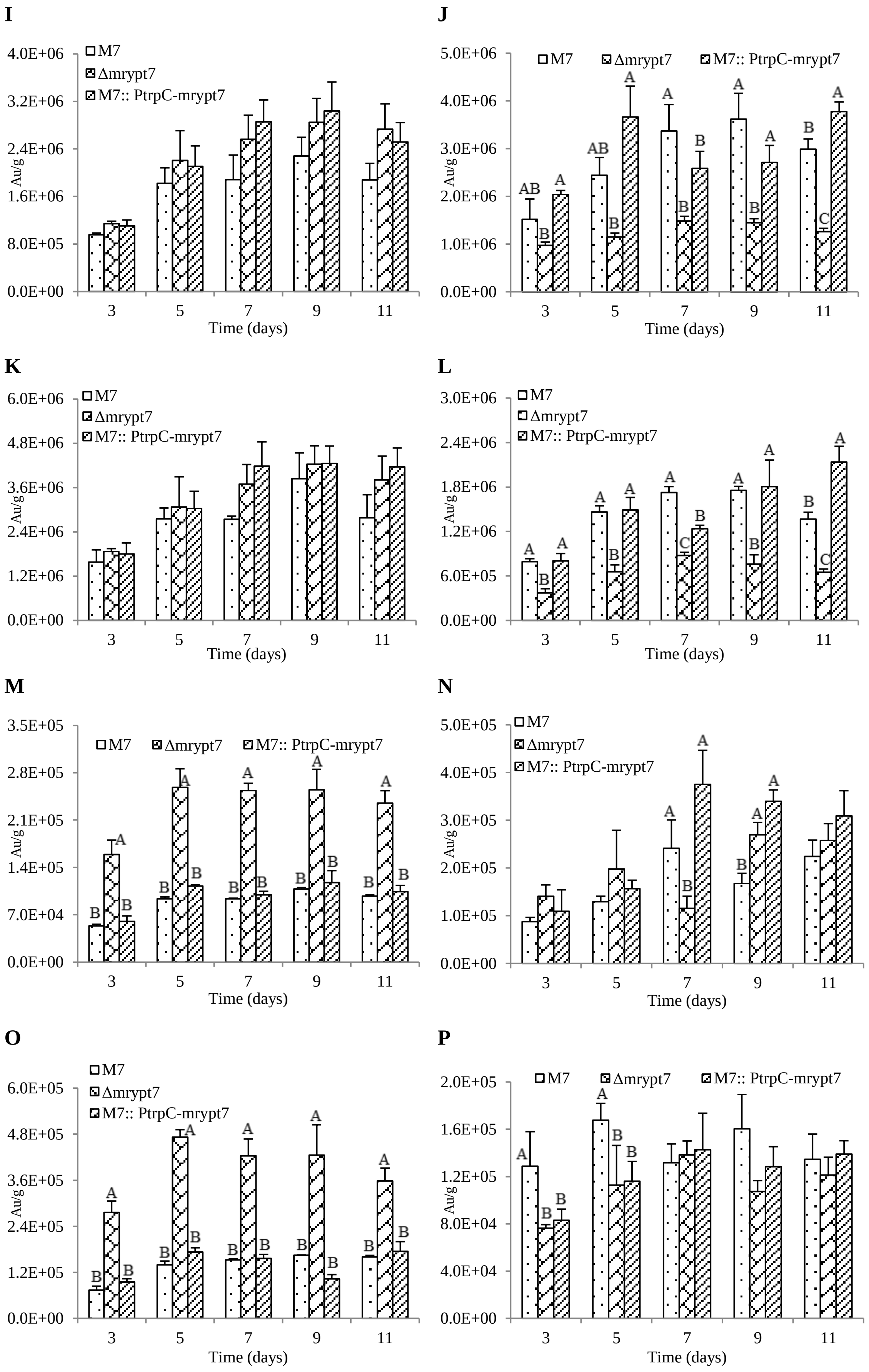

I
M7
Δmrypt7
M7:: PtrpC-mrypt7
Au/g
4.0E+06
3.2E+06
2.4E+06
1.6E+06
8.0E+05
0.0E+00
3
5
7
9
11
Time (days)
J
M7
Δmrypt7
M7:: PtrpC-mrypt7
Au/g
5.0E+06
4.0E+06
3.0E+06
2.0E+06
1.0E+06
0.0E+00
AB
B
A
AB
B
A
A
B
B
A
B
A
B
C
A
3
5
7
9
11
Time (days)
K
M7
Δmrypt7
M7:: PtrpC-mrypt7
Au/g
6.0E+06
4.8E+06
3.6E+06
2.4E+06
1.2E+06
0.0E+00
3
5
7
9
11
Time (days)
L
M7
Δmrypt7
M7:: PtrpC-mrypt7
Au/g
3.0E+06
2.4E+06
1.8E+06
1.2E+06
6.0E+05
0.0E+00
A
B
A
A
B
A
A
C
B
A
B
A
B
C
A
3
5
7
9
11
Time (days)
M
M7
Δmrypt7
M7:: PtrpC-mrypt7
Au/g
3.5E+05
2.8E+05
2.1E+05
1.4E+05
7.0E+04
0.0E+00
B
A
B
B
A
B
B
A
B
B
A
B
B
A
B
3
5
7
9
11
Time (days)
N
M7
Δmrypt7
M7:: PtrpC-mrypt7
Au/g
5.0E+05
4.0E+05
3.0E+05
2.0E+05
1.0E+05
0.0E+00
A
B
A
B
A
A
3
5
7
9
11
Time (days)
O
M7
Δmrypt7
M7:: PtrpC-mrypt7
Au/g
6.0E+05
4.8E+05
3.6E+05
2.4E+05
1.2E+05
0.0E+00
B
A
B
B
A
B
B
A
B
B
A
B
B
A
B
3
5
7
9
11
Time (days)
P
M7
Δmrypt7
M7:: PtrpC-mrypt7
Au/g
2.0E+05
1.6E+05
1.2E+05
8.0E+04
4.0E+04
0.0E+00
A
B
B
A
B
B
3
5
7
9
11
Time (days)

Fig. S4 *Monascus* pigments yield analysis of *M.ruber* M7, Δ*mrypt*7 and M7::*PtrpC-mrypt*7. (A) The yield of intracellular Monasfloure A. (B) The yield of extracellular Monasfloure A. (C) The yield of intracellular Monascine. (D) The yield of extracellular Monascine. (E) The yield of intracellular Monasfluore B. (F) The yield of extracellular Monasfluore B. (G) The yield of intracellular Ankaflavin. (H) The yield of extracellular Ankaflavin. (I) The yield of intracellular Rubropunctatin. (J) The yield of extracellular Rubropunctatin. (K) The yield of intracellular Monascuburin. (L) The yield of extracellular Monascuburin. (M) The yield of intracellular Rubropunctamine. (N) The yield of extracellular Rubropunctamine. (O) The yield of intracellular Monascuburamine. (P) The yield of extracellular Monascuburamine. The error bar represents the standard deviation between the three repeats. Capitals signify p-value <0.01.
