# Supplementary material for: A Comprehensive Analysis of the Small GTPases Ypt7 Involved in the Regulation of Fungal Development and Secondary Metabolism in Monascus ruber M7
Source: Front Microbiol. 2019 Mar 18;10:452. doi: 10.3389/fmicb.2019.00452 (PMC6431638; doi:10.3389/fmicb.2019.00452)
Supplement: Table S1 — Ypt homologous genes in the M. ruber M7 genome. [file Table_1.DOCX]

**Table S1 Ypt homologous genes in the *M. ruber* M7 genome**

| Ypt group  (Fungi) | Gene ID  (*M. ruber* M7 genome ) | Homologous fungi (Accession) | Identity(%) |
| --- | --- | --- | --- |
| Ypt1/ YptA | GME380 | *A.kawachii*  (GAA88155.1) | 92 |
| Ypt2 | GME1186 | *A.niger*  (XP_001400630.1) | 96 |
| Ypt3/ Ypt8 | GME7826 | *A.steynii*  (XP_024700564.1) | 94 |
| Ypt4 | GME1936 | *A.fumigatus*  (XP_746678.2) | 77 |
| Ypt5/ Ypt10 | GME8000 | *A.clavatus*  (XP_001271697.1) | 93 |
| Ypt6/ Ypt11 | GME2006 | *A.fumigatus*  (XP_755892.1) | 99 |
| Ypt7 | GME5756 | *A.fischeri*  (XP_001259484.1) | 91 |
|  |  | *A. oryzae*  (XP_001824054.1) | 91 |
|  |  | *A. niger*  (XP_001398680.2) | 91 |
|  |  | *P. oxalicum*  (EPS32522.1) | 91 |
|  |  | *P. zonata*  (XP_022585464.1) | 91 |
|  |  |  |  |
