# Supplementary material for: A Comprehensive Analysis of the Small GTPases Ypt7 Involved in the Regulation of Fungal Development and Secondary Metabolism in Monascus ruber M7
Source: Front Microbiol. 2019 Mar 18;10:452. doi: 10.3389/fmicb.2019.00452 (PMC6431638; doi:10.3389/fmicb.2019.00452)
Supplement: Table S2 — Primers used for the deletion and overexpression of mrypt7 gene. [file Table_2.docx]

**Table S2 Primers used for the deletion and overexpression of mrypt7 gene**

| Primers | Sequences (5’→3’) | Functions |
| --- | --- | --- |
| Y7-Zup1 | CGGTCAATAAAGTCGCAGA | For amplification of the 522 bp of the 5’ flanking regions of the *mrypt7* gene for the deletion cassete |
| Y7-Zdo1 | GGCTACGGTTCGATGGGGTTGAGTTGGGGTAGGTTGACTTCGTTGAGA |  |
| Y7-Yup1 | CATGCATGTTGCATGATGATGTCTTCG  GTTCACATCTGATAC | For amplification of the 684 bp of the 3’ flanking regions of the *mrypt7* gene |
| Y7-Ydo1 | GACATTAGCGATAACTGGTCATC |  |
| G418up | CCAACTCAACCCCATCGAACCGTAACC | For amplification of the 1221 bp of the *G418* gene from the plasmid pKN1 was used as probe 2 |
| G418do | ATCATCATGCAACATGCATG |  |
| Y7-up1 | GTGATTCTGGGTGATAGTGGT | For amplification of the 664 bp of the partial *mrypt7* gene which was used as probe 1 |
| Y7-do1 | CGACGTTGACGGCTTCTTT |  |
| Y7-Zup2 | GGACATCTAAGACCAATCACAA | For amplification of the 949 bp of the 5’ flanking regions of the *mrypt7* gene for the overexpression cassete |
| Y7-Zdo2 | *GGTTACGGTTCGATGGGGTTGAGTTGG*  GGTAGGTTGACTTCGTTGAGA |  |
| PtrpCup | GTGGACAGAAGATGATATTG | For amplification of the 373 bp of the *trpC* promotor from the plasmid pSKH |
| PtrpCdo | CATATCGATGCTTGGGTAGAATA |  |
| Y7-up2 | *TATTCTACCCAAGCATCGATATG*  CCGCACATCTACCGAAGGAAC | For amplification of the 1982 bp of the *mrypt7* gene for the overexpression cassete |
| Y7-do2 | GACATTAGCGATAACTGGTCAT |  |
