# Supplementary material for: A Comprehensive Analysis of the Small GTPases Ypt7 Involved in the Regulation of Fungal Development and Secondary Metabolism in Monascus ruber M7
Source: Front Microbiol. 2019 Mar 18;10:452. doi: 10.3389/fmicb.2019.00452 (PMC6431638; doi:10.3389/fmicb.2019.00452)
Supplement: Table S3 — The proposed genes involved in vesicle transport. [file Table_3.docx]

**Table S3 The proposed genes involved in vesicle transport**

| Gene ID | Discription | | Up/Down regulation* | | | |
| --- | --- | --- | --- | --- | --- | --- |
|  |  |  | M7-3d  *vs*  M7-7d | △*mrypt*7-3d  *vs*  △*mrypt*7-7d | M7-3d  *vs*  △*mrypt*7-3d | M7-7d  *vs*  △*mrypt*7-7d |
| GME2157 | SNARE | Bet1 | - | Down | Up | - |
| GME67 |  | Bos1 | - | - | Up | Up |
| GME2292 |  | Stx7 | - | - | Up | - |
| GME5065 |  | Stx16 | - | - | Up | - |
| GME3522 | Tethering factor | p115-golgins | - | - | - | Down |
| GME783 |  | Vacuolar protein sorting | Up | Up | - | Up |
| GME5186 |  | Transport protein particle (TRAPP) complex | - | - | Up | - |
| GME380 | Ypt1 | GTP-binding protein ypt1 | down | down | - | - |
| GME1186 | Ypt2 | GTP-binding protein ypt2 | - | - | - | - |
| GME7826 | Ypt3 | Small G proteins family | - | - | - | up |
| GME1936 | Ypt4 | GTPase related small G proteins | - | - | - | - |
| GME8000 | Ypt5 | Rab GTPase Ypt5 | - | - | - | - |
| GME2006 | Ypt6 | Ras small monomeric GTPase Rab6 | - | - | - | - |

*Significantly different expression was identified by NOISeq method with an absolute value of log_2_-fold change >1 and Probability>0.8

“Up” means the gene was up-regulated in the sample set; “Down” means the gene was down-regulated in the sample set;

“-” means the gene possessed similar expression level in the sample set.
